# *PIEZO1* variants that reduce open channel probability are associated with familial osteoarthritis

**DOI:** 10.1016/j.jbc.2026.111426

**Published:** 2026-04-02

**Authors:** Michael J. Jurynec, Elena Nosyreva, David Thompson, Kendra A. Novak, Derek J. Matheson, Shivakumar R. Veerabhadraiah, Ying Ma, Matthew C. Smith, Nikolas H. Kazmers, Stefan Feier, Ghanim Ullah, Ruhma Syeda

**Affiliations:** 1 Department of Orthopaedics, University of Utah, Salt Lake City, Utah, USA; 2 Department of Human Genetics, University of Utah, Salt Lake City, Utah, USA; 3 Department of Biomedical Engineering, University of Utah, Salt Lake City, Utah, USA; 4 Department of Neuroscience, UT Southwestern Medical Center, Dallas, Texas, USA; 5 Department of Physics, University of South Florida, Tampa, Florida, USA

**Keywords:** calcium channel, cartilage biology, genetic polymorphism, genetics, mechanically activated channels, mechanotransduction, osteoarthritis gene, osteoarthritis, PIEZO1

## Abstract

Synovial joints respond to physical forces to maintain tissue homeostasis. Disruption of joint homeostasis results in the development of osteoarthritis (OA), a disease characterized by abnormal remodeling of joint tissues. PIEZO1 is a mechanosensitive cation channel in the joint directly regulated by mechanical stimulus. To test whether PIEZO1 is associated with OA susceptibility, we determined whether variants affecting *PIEZO1* are associated with age-associated familial OA. We identified four rare coding variants affecting *PIEZO1* that are associated with dominant familial OA. Single-channel analysis demonstrated that all PIEZO1 mutant channels act in a dominant-negative manner to reduce the open probability of the channel in response to pressure. We show that a GWAS mutation in *PIEZO1* associated with reduced joint replacement results in increased channel activity. The familial and GWAS alleles have differential effects on gene expression in primary chondrocytes and synovial fibroblasts. Our data support the hypothesis that reduced PIEZO1 activity confers susceptibility to age-associated OA, whereas increased PIEZO1 activity may be associated with reduced OA susceptibility.

The synovial joint is a mechanosensitive organ that responds to various physical forces, including compressive, shear, and hydrostatic stresses (1, 2). Cells of the joint sense and respond to daily physical forces to maintain homeostasis of the organ. Disruption of mechanosensing or mechanotransduction can lead to molecular and cellular changes in the joint that are associated with the development of osteoarthritis (OA) (3, 4, 5, 6, 7), which is characterized by loss of joint space, degeneration of articular cartilage, remodeling of bone and other joint tissues, low-grade inflammation, and pain (8, 9). Many physiological and environmental risk factors are associated with increased risk of developing OA, including genetics, traumatic joint injury, aging, obesity, and altered biomechanics. Mechanosensing and mechanotransduction can be influenced by many of the same factors, which can be exacerbated by mechanical overloading, injury, or unloading (10, 11, 12, 13, 14). Despite the importance of mechanobiology in joint homeostasis and disease, we do not fully understand how alterations of the proteins that sense and respond to physical forces contribute to the development of OA, including in weight-bearing vs non-weight-bearing joints (15, 16).

PIEZO1 and TRPV4 are the key mechanosensitive cation channels in the joint directly regulated by mechanical stimulus. Previous *in vitro* studies using chondrocytes (the major cell type of cartilage) have indicated that PIEZO1 is activated at high levels of cellular deformation (*e*.*g*. hyperphysiological load such as a traumatic injury), and TRPV4 is activated under normal physiological load (17, 18, 19, 20, 21). Upon activation of PIEZO1, there is an influx of Ca^2+^ and Na ^+^ into the cell which leads to cell volume changes, activation of signaling pathways, and changes in gene expression (4, 5, 18, 19, 22, 23). Activation of PIEZO1 in the joint has been proposed to promote the OA phenotype by inducing inflammatory and catabolic gene expression, cell death, and senescence (3, 17, 18, 23, 24). In line with this, i*n vivo* studies using a nonspecific PIEZO1 antagonist (GsMTx4) suggests that inhibition of PIEZO1 is protective against injury-induced OA in rodent models (25, 26). In contrast to PIEZO1, TRPV4 activation in the joint is largely considered necessary to maintain homeostasis of the joint through activation of anabolic gene expression (21, 23, 27, 28). While previous studies have implicated hyperphysiological activation of PIEZO1 to elicit expression of OA-associated markers, a recent study has demonstrated that activation of PIEZO1 with a chemical agonist promotes expression of prochondrogenic genes and increased deposition of sulfated glycosaminoglycans (23), and another study showed activation of PIEZO1 in a subset of superficial tibial chondrocytes is necessary for protection against injury induced OA (29). These data suggest that PIEZO1 may have context-dependent or tissue-specific roles in maintaining homeostasis of the joint.

Despite the significant genetic contribution to OA (30, 31, 32, 33, 34), there is only one reported genetic association of *PIEZO1* variants with the OA phenotype (35). A genome-wide association study (GWAS) identified a dominant rare coding allele of *PIEZO1* that was associated with reduced OA progression (35). While this allele was associated with reduced OA progression (as defined by joint replacement), it is unknown how this mutation affects PIEZO1 channel function. No molecular studies were performed to determine if this mutation alters PIEZO1 channel activity. Therefore, we cannot determine if reduced or increased PIEZO1 mutant activity is associated with reduced OA progression in humans. In sum, the published data indicate that the role of PIEZO1 in OA is not resolved and that the channel may have context dependent or tissue-specific roles.

The genetic analysis of families with dominant forms of OA allows us to identify highly penetrant coding alleles that have a determinate effect on promoting OA, independent of prior biases on how protein activity may affect the OA phenotype or the tissue-specific requirement of the mutant alleles (36, 37, 38, 39, 40, 41, 42, 43, 44, 45). Non-null alleles further allow for functional studies to determine how the coding mutations affect protein activity and function in a physiologically relevant context. For example, compound heterozygous coding variants of *PIEZO1* were discovered in an individual with Prune Belly Syndrome (46). Extensive single-channel functional analyses demonstrated that the *PIEZO1* mutations associated with Prune Belly Syndrome reduced the pressure-induced open probability of the channel (46). Here, we report the identification of four families with age-associated OA with independent OA-susceptibility alleles in *PIEZO1*. All four familial *PIEZO1* mutant channels have reduced activity, while the GWAS mutation results in increased channel activity when compared with WT. The familial and GWAS alleles have differential effects on gene expression in primary human chondrocytes and primary mouse synovial fibroblasts. Our data suggest that reduced PIEZO1 channel activity increases susceptibility to age-associated OA, and increased PIEZO1 channel activity may be protective in the absence of acute injury. We hypothesize that PIEZO1 may have context-dependent or tissue-specific roles in injury vs aging.

## Results

### Identification of *PIEZO1* variants in families with age-associated OA

We took an unbiased genetic approach to determine if mutations in *PIEZO1* have a strong effect on OA susceptibility by utilizing a large statewide medical genetics database, the Utah Population Database (36, 37, 39, 47). We identified a cohort of 151 families with dominant forms of non-syndromic familial OA (including both weight-bearing and non-weight-bearing joints) that were free from acute or traumatic joint injury and other potentially confounding comorbidities (*e*.*g*., rheumatoid arthritis) (Supporting Information) (36, 37, 38, 39). Each family is characterized by a defining form of OA that affects a distinct subset of joints, including erosive hand OA (EHOA) (39, 48, 49) and interphalangeal (IP) joint OA(50, 51). Hand OA is a common, heterogeneous form of OA that affects females more than males (39, 50, 51, 52, 53, 54). Although families are identified by a shared OA phenotype, individuals in a family often have OA in additional joints, including the large weight-bearing joints such as the hip, knee, and spine (Table 1), which may reflect the necessity of PIEZO1 activity in all joints to maintain homeostasis.

**Table 1:** Osteoarthritis families and phenotype details

Family ID	Individual/sex/relation to proband	Joints affected	Cardiac/Blood phenotypes	Bone/MSK phenotypes
IP joint OA 53529	I/F/proband	Bilateral distal interphalangeal (IP) and EHOA		Localized decreased bone mineral density (hand)
	II/M/son	Carpometacarpal (CMC), metacarpophalangeal, bilateral IP, hip, and shoulder		
	III/F/sister	IP		
	IIII/M/husband	Unaffected		
IP joint OA 54034	I/M/proband	IP, ankle, spine, knees, and elbow	Sinus node dysfunction, Atrial fibrillation, hypertension	Olecrenon fracture, burst fracture of lumbar vertebra, osteoporosis, intervertebral disc degeneration
	II/F/daughter	IP, CMC, and spine	Heart murmur	Osteoporosis, fibula fracture, intervertebral disc degeneration
	III/F/daughter	Unaffected		
EHOA 13	I/F/proband	EHOA, hip, knee, spine, ankle	Anemia, hypertension	Osteoporosis, degenerative disc disease, toe fracture
	II/M/brother	EHOA	Atrial fibrillation, hypertension	
	III/M/brother	EHOA		Gouty arthropathy of knee
EHOA 15	I/F/proband	EHOA, distal IP, CMC, knees, cervical spine		Degenerative disc disease, osteopenia
	II/F/maternal aunt	EHOA		
	III/F/mother	EHOA		

Detailed medical chart review of the OA families. Individuals identified with EHOA or IP joint OA also have other affected joints (*e*.*g*., hip, knee, and ankle). Individuals identified with EHOA or IP joint OA also have cardiac/blood, or bone/musculoskeletal (MSK) phenotypes associated with altered PIEZO1 activity.

**Figure 1:**
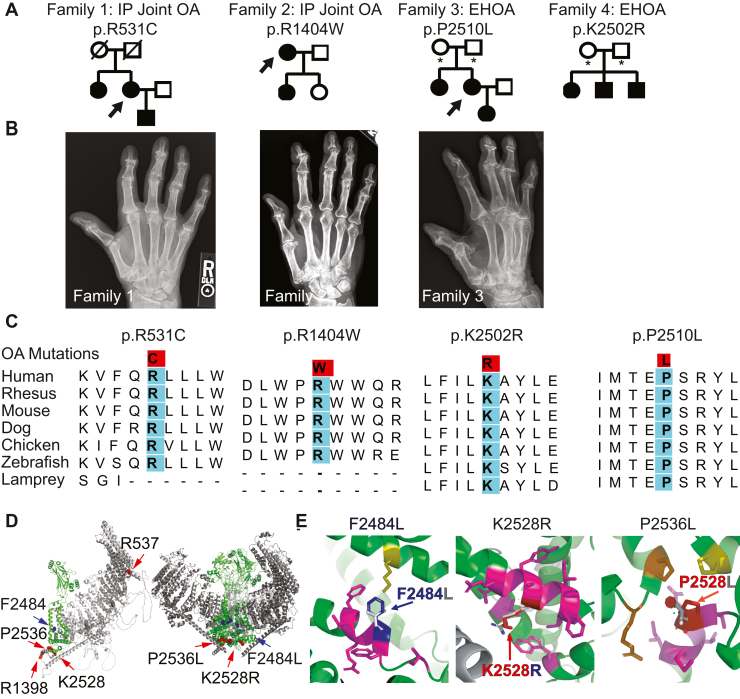
**Dominant mutations in *PIEZO1* are associated with hand OA**. *A*, interphalangeal (IP) joint OA and erosive hand OA (EHOA) segregate as apparent dominant traits in four independent families. The *PIEZO1* mutation identified in each family is noted above the pedigree. Circles = females, squares = males, slash = deceased. Filled circles/squares = affected individuals; open circles/squares = individuals with no known history of hand OA. Asterisks = individuals with an unknown hand OA diagnosis. *B*, hand radiographs of individuals marked with an arrow in the high-risk pedigrees. *C*, sequence alignment of the amino acids around the OA-associated *PIEZO1* mutations. The amino acids mutated in the OA families (in *red*) are invariant in vertebrates from human through lamprey. WT amino acids are highlighted in *cyan*. For two of the alleles, we were unable to identify high quality sequence for lamprey (p.R531C) and zebrafish and lamprey (p.R1404W) and thus these positions are missing from the alignment. *D*, AlphaFold2 PIEZO1 predicted monomer structure (*left*) and Cryo-EM based mouse PIEZO1 trimeric structure (PDB ID #6B3R) (*right*). PIEZO1 blades (*grey*) encompass residues 1 to 2109. Pore domain (*green*) spans from residues 2110 to 2547. Familial mutations (mouse amino acids) are shown in red and the GWAS mutation in *blue*. Human mutations and corresponding mouse amino acids in parenthesis: p.R531C (p.R537), p.R1404W (p.R1398), p.K2502R (p.K2528), p.P2510L (p.P2536), p.F2458L (p.F2484L). *E*, insets of structurally resolved regions (PDB ID #6B3R) indicating residues within 4 Å whose sidechains may interact with the WT residues. *Blue* and *red* mutation loci are indicated on the first chain of the trimer, with magenta residues indicating interacting partners on the first chain, *orange* indicating second chain residues, and *yellow* third chain residues.

We performed whole exome sequence analysis on informative members of families and identified coding variants that invariably segregated with OA (37, 38). We identified rare *PIEZO1* coding variants in four independent families diagnosed with bilateral EHOA or IP joint OA (Fig. 1, *A* and *B* and Table 1). Among the analyzed family members, *PIEZO1* variants were carried by all individuals with OA and absent from disease-free individuals, indicating the alleles act dominantly and appear to be completely penetrant. All variants were evolutionarily conserved in vertebrates (Fig. 1*C*) and rare in the general population (minor allele frequency < 0.01) (Table 2). The allele frequencies for p.R531C, p.R1404W, p.K2502R, and p.P2510L, in the Genome Aggregation Database (gnomADv2.1.1) are 0.0004740, 0.0007266, 0.006454 and 0.006832, respectively (Table 2). All four variants were predicted to be damaging or disease causing based on PolyPhen-2 (55) and MutationTaster analysis (56). An independent analysis using CADD (57) demonstrated that the p.R531C and p.R1404W variants are ‘likely deleterious’ (CADD score of ≥ 30) (Table 2). When mapped onto the mouse PIEZO1 Cryo-EM structure, the mutations are in key functional regions of the protein (Fig. 1, A and B and Table 2). Two mutations, p.K2502R and p.P2510L, are in the PIEZO1 pore domain (58), and p.R531C is in the peripheral blade region and is predicted to be near the inner leaflet of the lipid bilayer. The p.R1404W mutation is in a structurally unresolved region just before the beam that converges near the intracellular mouth of the channel and in proximity to the mutations in the pore (Fig. 1, *D* and *E*). In silico mutagenesis predictions identify the rotamers and degree of strain for three structurally resolved residues and respective mutations. Three rotamers of p.F2484L were predicted, with degrees of strain ranging from 12.6 (least energetically unfavorable) to 15.5. p.K2528R is predicted to have 24 rotamers with strain ranging from 7.4 to 45.3. p.P2536L is predicted to have three rotamers, ranging from 21.1 to 26.7 degrees of strain (Supplemental Movies 1–3). The least energetically unfavorable (lowest strain) rotamers are represented in the mutation insets’ still images Figure 1*E*.

**Table 2:** *PIEZO1* variants identified in independent osteoarthritis families

OA phenotype (family)	Human variant (mouse amino acid)	rsID/Minor allele frequency	PolyPhen-2 and MutationTaster/CADD score
IP joint OA 53529	p.R531C (p.R537)	rs146505418/0.0008618	Damaging/32.0
IP joint OA 54034	p.R1404W (p.R1398)	rs188966111/0.0006523	Damaging/32.0
EHOA 13	p.K2502R (p.K2528)	rs34830861/0.005809	Damaging/27.5
EHOA 15	p.P2510L (p.2536)	rs61745086/0.00839	Damaging/27.5

The variants, allele frequencies, and coding changes are indicated for each independent family. PolyPhen-2 and MutationTaster were used to determine if the mutation is predicted to be benign or damaging. CADD scores of ≥ 30 are considered ‘likely deleterious.

Given the importance of PIEZO1 function in multiple tissues and organ systems (5, 59, 60) and the association of *PIEZO1* mutations with several diseases (61), we analyzed the OA families for additional phenotypes related to altered PIEZO1 function. Dominant gain-of-function *PIEZO1* mutations are associated with dehydrated hereditary stomatocytosis (DHS) (62, 63, 64, 65, 66, 67). Recessive loss-of-function *PIEZO1* mutations are associated with congenital lymphatic dysplasia and Prune Belly Syndrome (46), while dominant loss-of-function mutations are associated with left ventricular outflow tract obstructions (LVOTO) (68). The p.K2502R and p.P2510L alleles have been identified in individuals diagnosed with DHS, but no functional studies were performed on these mutations to demonstrate that they are gain-of-function alleles (64, 65, 67). We did not note DHS or lymphatic dysplasia phenotypes in any of the affected OA family members (Table 1). The p.K2502R mutation was one of three *PIEZO1* mutations (p.Y2022H and p.S217L were the other two mutations) identified in individuals with LVOTO (68). Functional studies using HEK cells overexpressing WT or mutant channel indicated that these mutations act as dominant negatives to reduce PIEZO1 channel activity (68). In the OA family segregating the p.K2502R mutation, 2/3 affected individuals have atrial fibrillation (Table 1). Furthermore, the affected individuals segregating the p.R1404W mutation also have atrial fibrillation, heart murmur, or sinus node dysfunction, all of which are known phenotypes associated with LVOTO (69, 70, 71, 72) (Table 1). While none of the additional phenotypes were completely penetrant, osteopenia and osteoporosis were present in some individuals in all four families and several other cardiac defects, including hypertension, were noted in individuals in two of the families (Table 1). Our genetic studies indicate a striking correlation between the inheritance of variants in *PIEZO1* and the occurrence of disease within families exhibiting EHOA and IP joint OA, and a potential association of phenotypes commonly linked to left ventricular outflow tract defects.

### Single-channel analysis of OA-associated PIEZO1 variants

**Figure 2:**
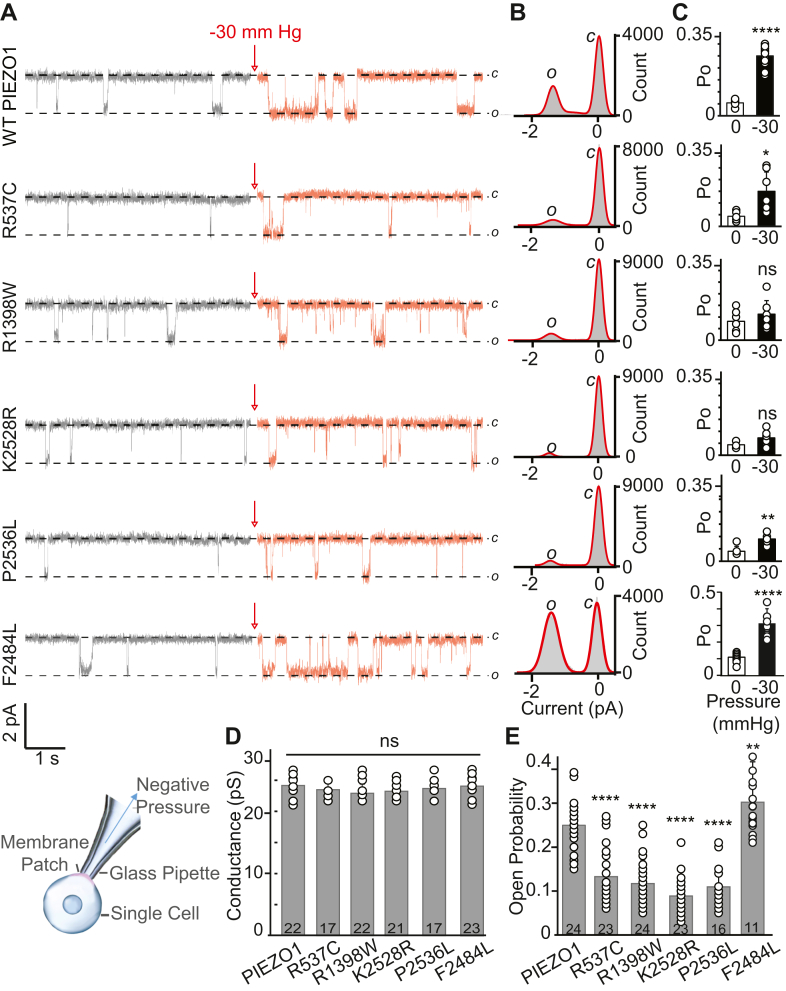
**Single-channel properties of WT and OA-associated PIEZO1 mutants obtained from HEK293T^ΔP1^ cell-attached recordings**. *A*, single-channel current recordings of WT and mutant proteins p.R537C, p.R1398W, p.K2528R, p.P2536L, and p.F2484L showing the closed (c) ∼0 pA and open (o) ∼ -1.5 pA state of the channel at −60 mV. The inset below depicts a cell-attached patch clamp configuration where glass pipette in contact with the cell forms the high resistance Giga Ohm seal for recording currents. Mechanical stimulus is applied to the membrane patch under the pipette tip in the form of negative pressure (*blue arrow*). The pipette solution contained 130 mM Na^+^ and 1 mM Ca^++^, the bath solution contained 140 mM K^+^ and 1 mM Mg^++^. *Red arrows* indicate application of −30 mm Hg pressure. *B*, all-point current amplitude histograms of the single-channel recordings shown in *panelA*. The area under the curve demonstrates the closed (c) and open (o) count of the relative states of the channel. X-axis is the current amplitude in picoamperes (pA). Y-axis is total counts. *C*, steady-state Open Probability (Po) of WT and mutant PIEZO1 with (*black bars*) and without (*white bars*) applied pressure (−30 mm Hg) to the patch of membrane. X-axis is pressure in mm Hg. Y-axis is single-channel Po. Experimental replica (n); WT n = 12, p.R537C n = 7, p.R1398W n = 8, p.K2528R n = 8, p.P2536L n = 6, and p.F2484L n = 11. The fold increase in the open probability after applied pressure; WT = 4.6, p.R537C = 3.5, p.R1398W = 1.4, p.K2528R = 1.7, R1398W, P2536L = 2.2, p.F2484L = 3 -fold. An unpaired two-tailed *t* test was used for comparison between the WT and mutant channels where, ∗*p* < 0.05, ∗∗*p* < 0.01, ∗∗∗∗*p* < 0.0001 and ns = not significant. *D*, single-channel conductance (pS) of WT and mutant PIEZO1 obtained from *panelB*, indicates no significant difference, n indicated in bar graph. *E*, steady-state Po of WT and mutant PIEZO1 acquired at −30 mm Hg with −60 mV, and calculated from the area under the curve from the open state. An unpaired two-tailed *t* test was used for comparison between the WT and each mutant channels where, ∗∗*p* < 0.01, ∗∗∗∗*p* < 0.0001. One-way ANOVA Post-Hoc test (Bonferroni correction) indicates that familial mutations p.R537C, p.R1398W, p.K2528R, p.P2536L are significantly different from WT PIEZO1, but no significant difference between these mutants themselves. GWAS mutation p.F2484L is significantly different from WT PIEZO1 and from all other mutants. ∗∗∗∗*p* < 0.0001 significant. Experimental replica numbers are shown in the bar graphs. The scatter bar graph plots (*C*, *D*, and *E*) represented as mean ± Standard Deviation.

The familial OA-associated *PIEZO1* mutations are predicted to be damaging (Table 2), but *in silico* predictions fail to determine if the mutations are gain- or loss-of-function. Furthermore, both gain- and loss-of-function *PIEZO1* mutations are associated with human disease phenotypes (5, 46). Previous *in vitro* studies indicated the p.K2502R allele is a loss-of-function mutation (68). Therefore, it is important to perform functional analysis to assess whether the mutations are causal and alter normal PIEZO1 function. We generated expression vectors encoding the mouse WT and five mutant PIEZO1 channels (4 familial and 1 GWAS mutant). The mouse amino acids corresponding to human mutations are indicated in Table 2. We expressed these constructs in HEK293T^ΔP1^ cells that are null for endogenous PIEZO1 activity to obtain and compare overexpressed WT and mutant channel function. In this context, normal WT PIEZO1 function refers to biophysical properties, such as permeation (single-channel conductance) and gating (open probability in response to applied pressure) properties obtained in HEK293T^ΔP1^ cells. All the mutants yielded single-channel activity in cell-attached mode (Fig. 2*A*), with no significant difference in single-channel currents obtained from all-point current-amplitude histograms (Fig. 2*B*). As expected for mechanosensitive channel, the open probability increases when direct pressure is applied to the patch of membrane expressing WT, familial mutants p.R537C and p.P2536L, and GWAS p.F2484L mutant. The fold increase in the open probability after applied pressure (−30 mmHg) was WT = 4.6, p.R537C = 3.5, p.P2536L = 2.2, p.F2484L = 3.2-fold (Fig. 2*C*). However, no significant difference was found before and after pressure application (−30 mm Hg) for the p.R1398W (fold change 1.4) and p.K2528R (fold change 1.7), (Fig. 2*C*). Moreover, each mutant was also tested at pressures ranging from −10 to −50 mm Hg to observe any significant trend. The open probability assayed at different pressures revealed no significant change in Po for p.R1398W, whereas p.K2528R showed significant increase at higher pressures only (−50 mm Hg) (Fig. S1). Together, these data suggest that the mechanosensitive gating properties are severely impaired in p.R1398W, and the channels did not respond to the range of pressures tested. Overall comparison of WT single-channel properties obtained from Current-Voltage relationship with that of OA-associated mutants clearly indicates no significant difference in unitary and slope conductance (Figs. 2*D* and S2). Unlike unitary and slope conductance, a sharp decrease in open probability of familial mutants to that of WT PIEZO1 was observed when assayed at −30 mm Hg pressure (Fig. 2*E*). In contrast, in the GWAS mutation, p.F2484L open probability was significantly higher than that of WT (Fig. 2*E*). The acquisition of single-channel data strongly suggests that mutants are expressed at the plasma membrane without any severe trafficking impairment, which is further confirmed by extracellular Myc-tag labeling of WT and mutant channels (Fig. S3). Together, these data indicate that the four familial *PIEZO1* mutations are hypomorphic (loss-of-function), whereas the GWAS mutation is hypermorphic (gain-of-function).

**Figure 3:**
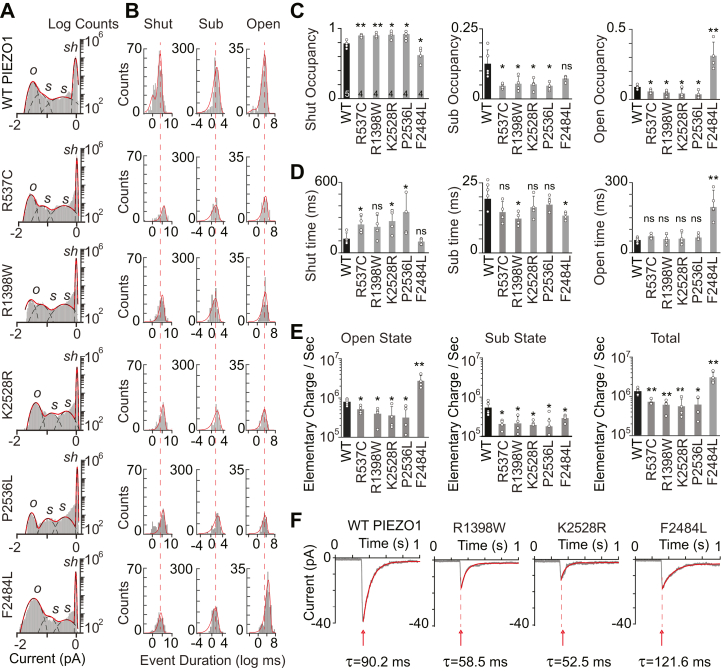
**Biophysical analysis of permeation and gating properties of WT PIEZO1 and OA-associated mutants**. *A*, single-channel current-amplitude histograms of WT and indicated mutations (*top* to *bottom*) showing the sub states (s) in between the shut state (sh) and Open state (o). Y-axis is event counts presented as logarithmic scale; X-axis is Current (pA). *B*, representative Dwell time histogram/distribution of PIEZO1 and mutants (*top* to *bottom* as in *panelA*) in Shut, Sub, and Open states. The *red dotted line* shows a visual comparison of peak shift of mutants when compared to WT PIEZO1. *C*, quantification and comparison of single-channel occupancy and (*D*) mean-lifetimes (*top* to *bottom*) in the indicated state. *E*, elementary charge per second (e/s) indicating ion flux/channel, calculated for individual open, sub, and total elementary charge for WT and indicated mutations. The scatter plot bar graph data in *C*, *D* and *E* (occupancy, mean-lifetimes and elementary charge) is represented as mean ± Standard Deviation of indicated experimental replicas (from *panelC*). Unpaired two-tailed t-tests (*C*–*E*) used for comparison between WT and individual mutation, ∗*p* < 0.05, ∗∗*p* < 0.01, not significant (ns). (*F*). Average of multiple channel traces (n = 5) of WT and indicated mutants exhibiting pressure activated PIEZO1 activity followed by inactivation, with inactivation time constant (τ) calculated by fitting single exponential curve. *Red arrow* indicates application of −30 mm Hg pressure.

Subconductance states are reported for WT PIEZO1 and considered as the inherent property of the channel (73). Here, we assess whether mutant channels also exhibit similar sub-level trends, despite having no significant change in single-channel conductance. All-point current-amplitude histograms constructed for all the mutants and fitted with the Gaussian function exhibit clear subconductance states that reside between the shut-state and open-state (Fig. 3*A*). Further, analysis shows that the occupancy and mean lifetime in different states varies between WT and mutant channels (Fig. 3, *B* and *C*). The shut-state occupancy of familial mutants was significantly higher than WT, but lower for GWAS mutant, signifying the loss-of-function and gain-of-function open probability trend (Fig. 3*C*). Similarly, the loss-of-function mutants exhibit significantly lower occupancy in sub-state than WT, whereas no significant change is observed between WT and GWAS sub-state. Importantly, the open-state occupancy was significantly lower for familial mutants and significantly higher for GWAS mutant. This analysis clearly shows loss-of-function behavior for familial mutants p.R537C, p.R1398W, p.K2528R, and p. P2536L, and gain-of-function trend for GWAS p.F2484L mutant when compared with WT PIEZO1.

Dwell time distribution was also compared between WT and mutant channels. The mean-lifetime spent in shut-state is significantly higher for three familial mutations p.R537C, p.K2528R, and p.P2536L, whereas mean lifetime in substate was significantly lower for p.R1398W and p.F2484L (Fig. 3*D*). No significant change in the mean-lifetime of open state is observed for the familial mutations, but the GWAS mutant was significantly higher. Since we calculated single-channel current in each state and occupancy in each state, we calculated elementary charge through the PIEZO1 channel (WT and mutants) when in sub-state and open-state (Fig. 3*E*). In line with our data presented above, the elementary charge through single-channel either in open or sub-state is significantly lower for all four familial mutants, and significantly higher for the GWAS mutant.

Based on our analysis (Fig. 3), we conclude that loss-of-function properties of all familial mutants depend on four distinct parameters; (1) increased shut-state occupancy, (2) decreased sub-state occupancy, (3) decreased open-state occupancy, and (iv) decreased elementary charge per second when the single-channel was in the sub or in the open-state. Of note is p.R1398W, which exhibits additional loss-of-function trend in terms of increased shut-state lifetime and decreased sub-state lifetime (Fig. 3, *C* and *D*). We also conclude that GWAS mutant p.F2484L exhibits gain-of-function properties when compared with WT PIEZO1 in terms of four distinct parameters; (1) decreased shut-state occupancy, (2) increased open-state occupancy, (3) increased open-state lifetime, and (4) increased elementary charge per second in open and sub-state at a single-channel level.

Based on our single-channel analysis, we chose two loss-of-function familial mutants (p.R1398W and p.K2528R) and the GWAS gain-of-function p.F2484L mutant to assess inactivation properties that cannot be studied at a steady-state single-channel level. Data were averaged from five to six distinct cells acquired at −30 mm Hg (Fig. 3*F*). As expected, mechanically activated currents were observed instantly with pressure application, followed by inactivation property as seen for PIEZO1 (74). Distinct inactivation time constants are calculated by single exponential curve fitting to the ensemble-averaged current traces derived from multiple single-channel recordings, τ inactivation for WT = 90.2 ms, whereas for p.R1398W = 58.5 ms (1.5-fold lower than WT), p.K2528R = 52.5 ms (1.7-fold lower than WT), and for p.F2484L = 121.6 ms (1.4-fold higher than WT). Importantly, these τ values represent the macroscopic relaxation time constant of the channel population rather than the mean open time determined from single-channel dwell-time analysis. The ensemble macroscopic data also follows a similar loss-of-function and gain-of-function trend, as observed from single-channel data analysis.

### OA-associated familial mutants are dominant negative

All the familial OA-associated *PIEZO1* alleles segregate dominantly with the OA phenotype. To recapitulate these pathophysiology relevant conditions in the cellular *in vitro* assays, we co-expressed WT PIEZO1 (fused with TdTomato) and the familial mutants (fused with GFP) in HEK293T^ΔP1^ cells. Only the cells exhibiting both red and green signals were selected for patch clamp recordings to ensure the expression of both the WT PIEZO1 (red) and OA-associated familial variants (green) within the same cell (Figs. S4 and S5 and Supporting Information). Structural studies showed that PIEZO1 is a homo-trimeric channel where three identical subunits assemble to form a central ion conduction pore (75, 76). To date, there is no structural information on heteromeric PIEZO channels; however, there are functional assays that suggest heteromeric PIEZO channels can exist in cells (17, 46, 77). The co-expression of two PIEZO1 constructs can potentially produce three major combinations of PIEZO1 channels; (1) Homo-trimers of WT PIEZO1, (2) homo-trimers of familial mutant channels, and (3) hetero-trimers of WT and mutant subunits (either in 2:1 or 1:2, WT: Mutant subunit ratios). Since WT and familial mutants (when expressed alone) exhibit significantly different single-channel open probability, occupancy, and mean lifetimes in each state when assayed at −30 mm Hg (Fig. 2*E*), we relied on pressure-dependent gating properties such as occupancy and dwell times spent in the shut, open, and sub-conductance state to assess whether co-expression produces more prevalent WT-like activity or mutant-like activity.

**Figure 4:**
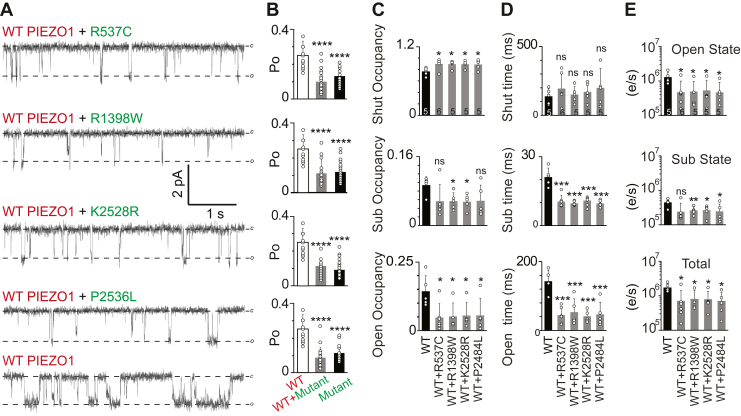
**Functional dominant negative effect of OA-associated PIEZO1 mutations**. *A*, single-channel current recordings of either WT, mutants, or WT/mutant co-expressed PIEZO1 channels obtained from HEK293T^ΔP1^ cells. WT PIEZO1 is tagged with TdTomato and mutant PIEZO1 is tagged with GFP. Co-expression of TdTomato and GFP is used to identify single cells expressing both WT and mutant channels. Data acquired at −60 mV and −30 mm Hg pressure. c = closed, o = open. *B*, Comparison of steady-state Po between WT, mutants, and WT/mutant co-expressed PIEZO1 channels. An unpaired two-tailed *t* test was used for comparison between the WT and each mutant channels where ∗∗∗∗*p* < 0.0001. One-way ANOVA Post-Hoc test (Bonferroni correction) indicates that Po for WT alone is significantly higher compared to all mutants and mutants co-expressed with WT PIEZO1. Experimental replica (n) between 13 and 27 individual patch recordings for each condition. *C*, Single-channel occupancy and (*D*) mean-lifetimes and (*E*) Elementary charge/second (e/s) of WT and WT co-expressed with mutants in Shut, Sub and Open state. Bar graphs (with indicated n) are presented as Mean ± Standard Deviation where unpaired two-tailed *t* test ∗*p* < 0.05; ∗∗*p* < 0.01, ∗∗∗*p* < 0.001, not significant (ns).

Co-expression of WT and mutant PIEZO1 produces activity that closely resembles the familial mutants' open probability and is significantly different from WT (*p* < 0.0001) (Fig. 4, *A* and *B*). No significant difference was found when the co-expression conditions were compared to the mutants expressed alone (*p* > 0.095), suggesting a functional dominant-negative effect of these mutations in HEK293T^ΔP1^ cells (Fig. 4). As expected from data obtained in single mutant expression, sub-states were observed in co-expressed conditions, with distinct occupancy in each state. Shut-state occupancy increased, while open-state occupancy decreased for all co-expressed mutants, albeit expressed with WT, signifying decreased open probability of the co-expressed mutant channels (Fig. 4*C*). Again, p.R1398W and p.K2528R exhibit strong effects when co-expressed with WT, as we observe a significantly decreased sub-state occupancy as compared to WT alone. Surprisingly, all co-expressed familial mutants follow the trend of decreased sub-state lifetime, decreased open-state lifetime and decreased elementary charge per second, when compared to WT alone (Fig. 4, *D* and *E*). This extensive analysis concluded that the mutants exhibit functionally dominant-negative trends even in the presence of WT subunits.

### Yoda1 potentiates loss-of-function OA-associated variants

**Figure 5:**
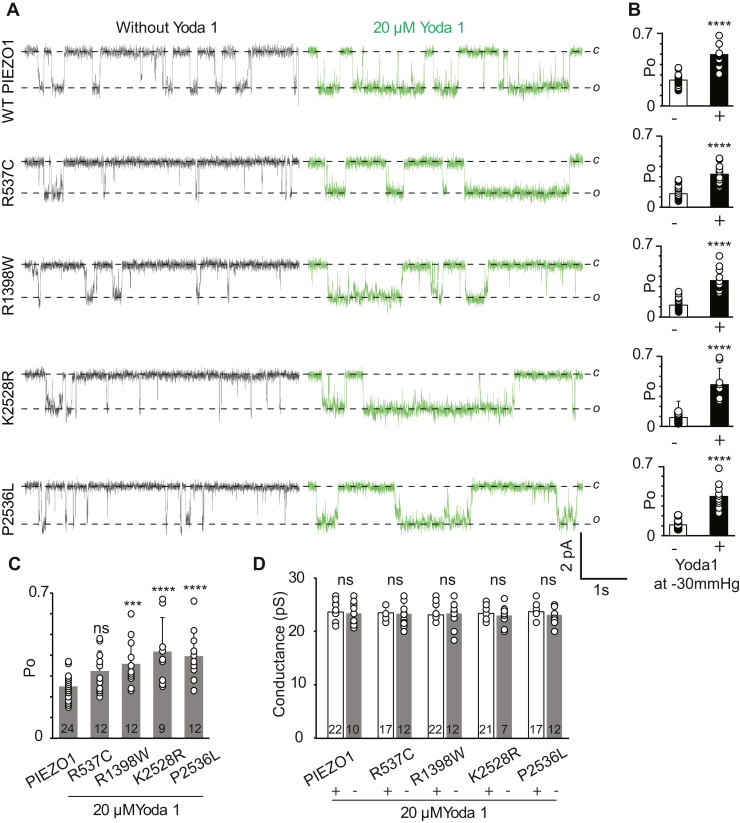
**Chemical modulation of WT and OA-associated PIEZO1 by Yoda1**. *A*, representative single-channel current recordings of WT and mutant PIEZO1 channels, obtained from cell-attached patches at −60 mV and −30 mm Hg pressure. *o* and *c* denote open and closed state of the channel. *B*, steady-state Po of WT and mutant PIEZO1 acquired at −30 mmHg and −60 mV with and without Yoda1. Unpaired two-tailed t-tests used for comparison between WT in the presence and absence of Yoda1, ∗∗∗∗*p* < 0.0001, (experimental replica; n between 9 and 26). *C*, comparison of steady-state Po of WT PIEZO1 (in the absence of Yoda1) and mutants (in the presence of Yoda1. One-way ANOVA Post-Hoc test (Bonferroni correction) indicates WT PIEZO1 Po is significantly different from p.R1398W (*p* < 0.001), p.K2528R, and p.P2536L (*p* < 0.0001). *D*, single-channel conductance of WT and mutant PIEZO1 channels measured with and without 20 μM Yoda1 in the pipette, not significant (ns) (*p* > 0.05). Bar graphs (with indicated n in *C* and *D*) are presented as Mean ± Standard Deviation.

Yoda1 is a PIEZO1 agonist that increases the open probability of the channel, delays inactivation, and allows calcium flux *via* PIEZO1 channels in the absence of applied forces (46, 78). Here, we tested the effect of Yoda1 on OA-associated loss-of-function PIEZO1 mutations by supplementing 20 μM Yoda1 in the patch pipette in cell-attached single-channel recordings (Fig. 5*A* and Supporting Information). As previously published, WT PIEZO1 responded to Yoda1 by exhibiting a 2-fold increase in open probability (46). Interestingly, all the familial mutants responded to Yoda1 with an increase in open probability as p.R537C = 1.5-fold, p.R1398W = 3-fold, p.K2528R = 4-fold, and p.P2536L = 2.5-fold (Fig. 5, *B* and *C*). Additionally, no change in the single-channel currents was observed with and without Yoda1 (Fig. 5*D*), suggesting that Yoda1 only changes the gating properties of the mutant channels. In summary, the functional assays showed that Yoda1 increases the open probability of all the familial mutants when compared with WT (Fig. 5*C*).

**Figure 6:**
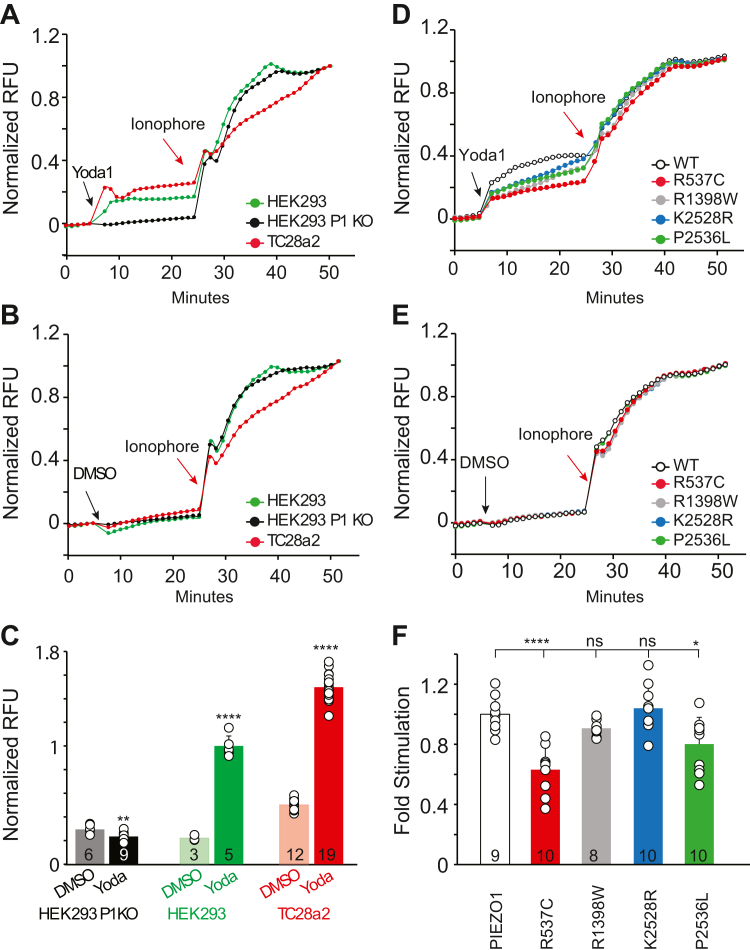
**Yoda1 specifically stimulates endogenous PIEZO1 inTC28a2chondrocytes, HEK293, and heterologous PIEZO1 in HEK293^ΔP1^ Cells**. *A–B*, fluorescent detection of intracellular calcium *via* Fluo-4 dye with or without Yoda1 (representative experiment) in the indicated native cells expressing endogenous PIEZO1, and pre-loaded with Fluo-4 before treatment with (*A*) 20 μM Yoda1 in 0.2% DMSO or (*B*) 0.2% DMSO control. *C*, Quantification of calcium indicated by normalized cell fluorescence induced by Yoda1. Yoda1 induces calcium in endogenously expressing cells but not in PIEZO1 knock-out cells. DMSO alone does not stimulate calcium influx in any of the cell lines, regardless of PIEZO1 endogenous expression. An unpaired two-tailed *t* test was used for comparison between DMSO and Yoda treatment in each cell type. Bar graphs (with indicated n) are presented as Mean ± Standard Deviation. *D–E*, Fluorescent detection of intracellular calcium *via* Fluo-4 with or without Yoda1 in HEK293^ΔP1^ cells over-expressing WT PIEZO1 or indicated OA-associated mutants (representative experiment), and pre-loaded with Fluo-4 before treatment with (*D*) 20 μM Yoda1 in 0.2% DMSO or (*E*) 0.2% DMSO control. *F*, data points reflect maximal activated signal prior to addition of ionophore (*red arrow*). Unpaired two-tailed t-tests used for comparison between WT and mutants, ∗*p* < 0.05, ∗∗∗*p* < 0.0001, ns (not significant). Addition of Yoda1 or DMSO at 5 min (*black arrows*). Ionophore A23187 (t = 24 min - *red arrows*) served as internal control to stimulate maximum non-specific calcium influx (*A–B* and *D–E*). RFU = Relative fluorescence units. Bar graphs (with indicated n in *C* and *F*) are presented as Mean ± Standard Deviation.

In addition to single-channel activity, we evaluated whole-cell calcium influx *via* a cell-based fluorescence assay using Fluo-4 dye. To determine the optimal cell line for these assays, we first established the presence of functional PIEZO1 between cell lines by assessing the endogenous expression and functionality of PIEZO1 in HEK293, HEK293 ^ΔP1^, and TC28a2 chondrocytes. Each cell line was pre-loaded with Fluo-4 dye and probenecid before treatment with 20 μM Yoda1 (solvent 0.2% DMSO) or 0.2% DMSO control (Fig 6, *A* and *B* and Fig. S6). Addition of Yoda1 stimulated calcium influx in the endogenously expressing PIEZO1 HEK293 and TC28a2 cells but failed to stimulate calcium influx in the HEK293 ^ΔP1^ cells (Fig. 6*A*). None of the three tested cell lines exhibited calcium influx upon addition of DMSO alone validating the specificity of Yoda1 to PIEZO1 (Fig. 6*B*). Furthermore, TC28a2 chondrocytes failed to achieve steady-state on addition of calcium ionophore within the timeframe tested (Fig. 6, *A* and *B*). Comparison of calcium influx signal for DMSO alone to Yoda1 for each of the three cell lines indicates significant stimulation from endogenously expressing PIEZO1 in HEK293 and TC28a2 upon addition of Yoda1, with the HEK293 cells having greatest fold stimulation, as TC28a2 cells exhibited higher background with DMSO alone samples (Fig. 6*C*). To avoid confounding effects of endogenous PIEZO1 expression when transiently over-expressing OA mutants and to ensure the assays achieve a true steady-state for calculation of maximal possible signal on addition of ionophore, we opted to assess the effects in the HEK293 ^ΔP1^ cell line.

HEK293^ΔP1^cells were transfected with either WT PIEZO1 or the familial OA mutants and pre-loaded with Fluo-4 dye and probenecid before treatment with 20 μM Yoda1 (solvent 0.2% DMSO) or 0.2% DMSO control (Fig. 6, *D* and *E*). In line with our previous data (46), addition of Yoda1 affected intracellular calcium levels by stimulating influx in cells expressing WT and the familial mutants, suggesting that all the mutant channels are chemically stimulated in the absence of applied mechanical forces (such as pressure, Figure 2, Figure 3, Figure 4, Figure 5) and are trafficked correctly to the membrane (Fig. S5). Treatment with DMSO alone did not induce calcium influx in cells transfected with WT or mutant PIEZO1 (Fig. 6*E*). Total cell fluorescence of p.R1398W and p.K2528R are not statistically different than WT PIEZO1, while p.R537C and p.P2536L achieved 63% and 80% of WT channel activity, respectively (Fig. 6*F*), indicating that the degree of calcium influx is dependent on the mutant expressed. In conclusion, using two functional assays, we show that the familial PIEZO1 variants’ gating properties can be modulated and stimulated by Yoda1. These experiments serve as proof of concept and demonstrate the possibility to discover small molecule modulators for OA-associated mutants that may become clinically relevant.

### The OA-associated *PIEZO1* variants alter gene expression in primary human chondrocytes

Our electrophysiological studies of WT and familial OA-associated PIEZO1 channels demonstrated that all four familial mutations are hypomorphic, while the GWAS allele appears to be hypermorphic. While there was not a significant difference in the steady-state open probability between the four familial mutant PIEZO1 channels, qualitatively, the p.R1398W (p.R1404W; human mutation) appears to be the most severe of the mutations (Figure 2, Figure 3 and Fig. S1). The p.R1398W mutation is located just before the beam that converges near the intracellular mouth of the channel, indicating it may affect the pore region (Fig. 1, *D* and *E*). Moreover, the family harboring the p.R1404W mutation exhibits the most extensive cardiac and bone/musculoskeletal phenotypes among the four families (Tables 1 and 2), suggesting that this allele may have the strongest effect *in vivo*. Given our single-channel results and the additional phenotypes in the family, we wanted to determine if the reduced electrophysiological activity of the p.R1398W mutant channel or the increased activity of the GWAS allele (p.F2484L) altered gene expression in primary human chondrocytes.

**Figure 7:**
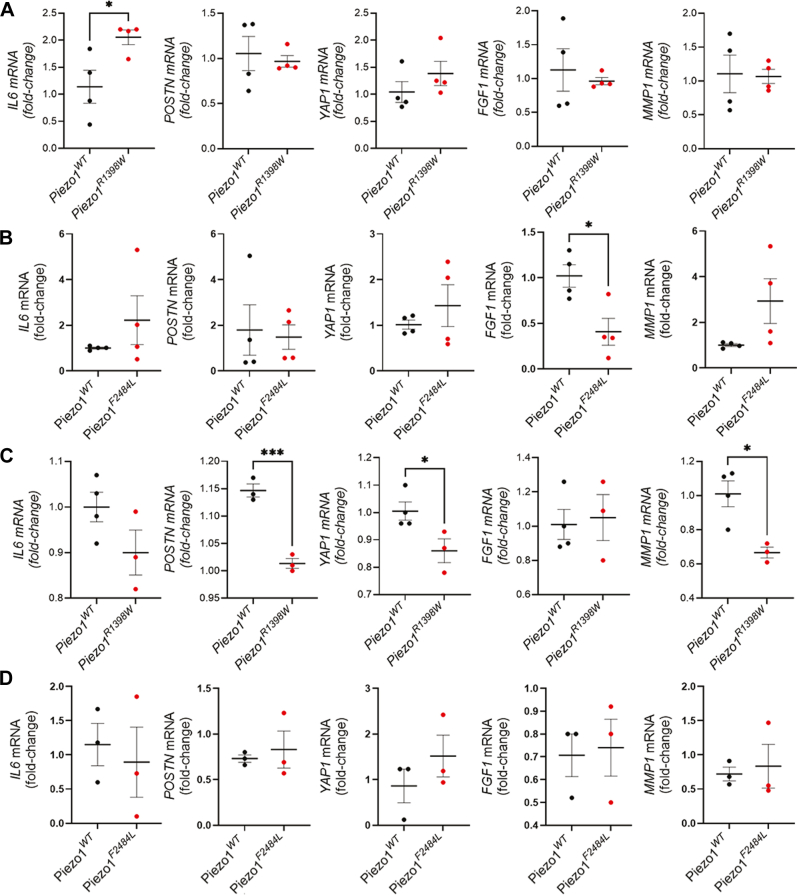
**Overexpression of *Piezo1*^*R1398W*^ or *Piezo1*^*F2484L*^ differentially effects the transcriptional response of primary human chondrocytes under control conditions or IL1β treatment**. RT-qPCR analysis of *IL6*, *POSTN*, *YAP1*, *FGF1*, and *MMP1* gene expression in primary human chondrocytes electroporated with *Piezo1*^*WT*^, *Piezo1*^*R1398W*^, or *Piezo1*^*F2484L*^. Gene expression in untreated chondrocytes (*A* and *B*) or chondrocytes treated with IL1β (10 ng/ml) for 24 h (*C* and *D*). Gene expression was normalized to *ACTB* and relative expression was calculated using the ΔΔCq method. mRNA levels are reported as fold-change (mean ± SEM) relative to *Piezo1*^*WT*^ electroporation. Statistically significant differences of *p* ≤ 0.05 (∗) and *p* ≤ 0.001 (∗∗∗) were determined by a two-tailed unpaired *t* test, n = 3 or 4 biological replicates as indicated in the scatter plots.

To determine if overexpression of the p.R1398W or p.F2484L mutant channels altered gene expression in chondrocytes, we electroporated primary human chondrocytes with constructs encoding WT (*Piezo1*^*WT*^), p.R1398W (*Piezo1*^*R1398W*^) or p.F2484L (*Piezo1*^*F2484L*^) and quantified changes in gene expression by RT-qPCR analysis (Fig. 7, Figs. S7–S9, and Supporting Information). We first tested if overexpression of *Piezo1*^*R1398W*^ or *Piezo1*^*F2484*^ could alter the expression of several genes associated with the OA phenotype. Compared with *Piezo1*^*WT*^, overexpression of *Piezo1*^*R1398W*^ in primary human chondrocytes resulted in an approximate 2-fold upregulation of the proinflammatory OA-associated cytokine, *IL6* (Fig. 7*A*) and overexpression of *Piezo1*^*F2484*^ resulted in the downregulation of *FGF1* (Fig. 7*B*). Overexpression of *Piezo1*^*R1398W*^ or *Piezo1*^*F2484*^ did not alter the expression of a selected subset of OA-associated genes (Fig 7, *A* and *B* and Fig. S9). These data indicate that overexpressing *Piezo1*^*R1398W*^ or *Piezo1*^*F2484*^ without mechanical or chemical activation of the channel had a differential effect on gene expression in primary human chondrocytes.

To test if reduced PIEZO1 activity changes the transcriptional response to an injury-induced cytokine, we treated primary human chondrocytes expressing *Piezo1*^*WT*^, *Piezo1*^*R1398W*^ or *Piezo1*^*F2484*^ with IL1β. IL1β expression in the synovial joint is associated with the OA phenotype and is used to induce a catabolic, injury-associated OA transcriptional response in cultured chondrocytes (79, 80, 81, 82, 83). IL1β or IL1α treatment of chondrocytes upregulates *PIEZO1* expression, and activation of PIEZO1 leads to upregulation of proinflammatory and catabolic gene expression (18, 84, 85). Considering this data and *in vivo* data indicating complete loss of PIEZO1 in articular chondrocytes reduced the severity of injury-induced OA (86, 87), we hypothesized that reduced PIEZO1 activity would decrease the chondrocyte catabolic response to IL1β stimulation. To test if overexpression of *Piezo1*^*R1398W*^ or *Piezo1*^*F2484*^ altered the chondrocyte transcriptional response to IL1β treatment, we electroporated primary human chondrocytes with constructs encoding *Piezo1*^*WT*^, *Piezo1*^*R1398W*^, or *Piezo1*^*F2484*^, treated the cells with IL1β for 24 h (10 μg/ml), and quantified changes in gene expression by RT-qPCR analysis. Compared with *Piezo1*^*WT*^, overexpression of *Piezo1*^*R1398W*^ in primary human chondrocytes treated with IL1β resulted in the downregulation *YAP1*, *MMP1*, and *POSTN* expression (Figs. 7*C* and S8), while overexpression of *Piezo1*^*F2484*^ had no effect on gene expression compared with WT (Figs. 7*D* and S8). These data indicate that the familial OA-associated *PIEZO1* mutations that reduce PIEZO1 channel activity have a functional effect on gene expression in an *in vitro* injury-associated model of OA.

### The OA-associated *PIEZO1* variants alter gene expression in primary mouse synovial fibroblasts

The joint is a complex organ in which multiple cells and tissues interact to maintain homeostasis (8). Changes in synovial cell composition and function are emerging as a critical driver of OA development (88, 89, 90). To determine if alteration of PIEZO1 activity changes the transcriptional response in a synovial cell, we overexpressed *Piezo1*^*WT*^, *Piezo1*^*R1398W*^, or *Piezo1*^*F2484L*^ in primary mouse synovial fibroblasts (SF) (90) with or without IL1β treatment and quantified gene expression by RT-qPCR analysis (Supporting Information). In the absence of IL1β stimulation, SF expressing *Piezo1*^*R1398W*^ show no changes in gene expression compared with *Piezo1*^*WT*^ cells (Fig. S10). In contrast, SF expressing *Piezo1*^*F2484L*^ upregulated expression of *Col10a1*, *Sox9*, and *Postn* in comparison with *Piezo1*^*WT*^ or *Piezo1*^*R1398W*^ expressing cells (Fig. S10). In response to IL1β treatment *Piezo1*^*WT*^ expressing SF upregulate many catabolic genes associated with OA development (Fig. S10). Expression of *Piezo1*^*R1398W*^ or *Piezo1*^*F2484L*^ altered the synovial fibroblast response to IL1β treatment. *Piezo1*^*R1398W*^ expressing cells upregulated expression of *Il1β*, *Ccl2*, *Mmp3*, *Yap*, *Postn*, and *p21* expression compared with *Piezo1*^*WT*^ cells, while cells expressing *Piezo1*^*F2484L*^ have increased expression of *Il6* and *Fgf1* (Fig. S10). Increased expression of *Il6* and *p21* was noted in *Piezo1*^*R1398W*^ expressing cells compared with cells expressing *Piezo1*^*F2484L*^ (Fig. S10). These data indicate that expression of the familial (p.R1398W) and the GWAS allele (p.F2484L) caused increased gene expression in primary mouse SF, with only the GWAS allele influencing unstimulated cells. While both alleles amplify the response to IL1β stimulation, the familial (p.R1398W) had a larger effect on gene expression. Collectively, our data suggests that the familial and GWAS alleles have context and cellular dependent roles in modulating gene expression in multiple cell types of the synovial joint.

## Discussion

PIEZO1 is a major mechanosensitive cation channel of the synovial joint that modulates the molecular and cellular response to various physical forces. Functional analyses of the familial OA-associated PIEZO1 proteins demonstrated that all four mutations are hypomorphic. In contrast with the familial mutations, we show at the molecular level for the first time that a previously identified GWAS *PIEZO1* mutation associated with reduced OA progression (35) is hypermorphic. The most parsimonious interpretation of the human genetic and electrophysiological data is that in the absence of acute or traumatic joint injury reduced PIEZO1 activity increases OA susceptibility, while increased PIEZO1 activity reduces OA susceptibility, although there are potential confounding differences between the familial and GWAS cohorts. The families we studied have well-defined EHOA or IP joint OA that occurs without acute or traumatic injury to the joint. The GWAS cohorts were identified based on knee and hip OA, and injury status was not described and the GWAS study was based on whether individuals with OA progressed to need a joint replacement. Although the human *PIEZO1* GWAS allele (p.F2458L) was associated with a reduction in joint replacement, no alleles of *PIEZO1* have been previously associated with OA susceptibility (48, 91, 92, 93, 94, 95, 96).

While our electrophysiological studies are important in determining how the OA-associated mutations directly alter PIEZO1 channel activity, they do not inform us about the impact these mutations have on the major mechanosensing cell of the synovial joint, the chondrocyte, or any other cell type of the joint organ. Several studies have clearly shown that PIEZO1 in chondrocytes, and to a lesser extent PIEZO2, functions to sense and respond to biomechanical forces and regulate a number of biological processes (3, 5, 17, 18, 19, 21, 22, 23, 24, 85). While many studies have focused on the role of PIEZO1 in chondrocytes, there is data to suggest it may function in other tissues to contribute to OA development, including fibroblast-like synoviocytes (97) and subchondral bone (98).

In the absence of hyperphysiological load or chemical activation of PIEZO1, overexpression of *Piezo1*^*R1398W*^ in primary human chondrocytes resulted in an approximate 2-fold upregulation of the proinflammatory OA-associated cytokine, *IL6* (Fig. 7*C*), while overexpression of *Piezo1*^*F2484L*^ resulted in the downregulation of the catabolic factor, *FGF1* (99, 100). This is consistent with our human genetic data and the hypothesis that reduced PIEZO1 activity in the absence of hyperphysiological load increases susceptibility to OA, while increased activity may have an anabolic effect. It has been recently demonstrated that chemical activation of PIEZO1 *in vitro* augments expression of expression of anabolic and pro-chondrogenic pathways (23). Data in mice indicates that deletion of *Piezo1* (86) or *Peizo1* and *Piezo2* (87) in articular chondrocytes protects against injury-induced OA. Based on this data, we hypothesize that the hypomorphic p.R1398W allele would be protective against injury-induced OA and the p.F2484L allele may exacerbate the catabolic response. Treatment of *Piezo1*^*R1398W*^ expressing primary human chondrocytes with IL1β to promote an acute, injury-associated OA-like transcriptional response resulted in downregulation of several genes associated with the OA-phenotype including *YAP*, *MMP1*, and *POSTN* (Fig. 7*C*). *POSTN* is a key mechanoresponsive gene involved in the induction of cellular senescence of nucleus pulpous cells and intervertebral disk degeneration. It’s expression in human nucleus pulpous cells is dependent on NF-κB, and inhibiting *POSTN* gene expression or activity prevented PIEZO1-dependent intervertebral disk degeneration development in a rat model (101). We did not see an effect of *Piezo1*^*F2484L*^ overexpression on gene expression in response to IL1β treatment compared with WT. It is possible that this allele only has a minor contribution to IL1β stimulation, needs to be mechanically activated, or regulates genes that were not included in our limited analysis.

We also examined if the familial allele (p.R1398W) and the GWAS allele (p.F12484L) could alter the transcriptional response in another cell type of the joint, the synovial fibroblast. Recent data indicates that PIEZO1 may have tissue specific roles in OA development (29). Only expression of the GWAS allele, *Piezo1*^*F2484L*^, was sufficient to alter gene expression in primary mouse synovial fibroblasts in the absence of IL1β, including upregulation of *Sox9*, *Col10a1*, *and Postn*. In response to IL1β treatment, *Piezo1*^*R1398W*^-expressing cells upregulated many proinflammatory and catabolic factors such as *Il1β*, *Ccl2*, *Mmp3*, *Yap*, *Postn*, and *p21* expression compared with *Piezo1*^*WT*^ expressing cells. *Piezo1*^*F2484L*^ had a smaller effect in response to IL1β as only *Il6* and *Fgf1* were upregulated. These data indicate that *Piezo1*^*R1398W*^ may have opposite effects in cartilage vs the synovium. This differential response may be due to intrinsic cellular differences such as mechanical load or downstream signaling pathways that modify how cells respond to PIEZO1 activity (3, 17, 18, 20, 23, 102, 103, 104). Supporting this hypothesis, PIEZO1 activity in a subset of superficial articular chondrocytes (Procr+) protects against injury induced OA and promotes cartilage regeneration following orthotopic transplantation (29). In sum, our data supports the hypothesis that PIEZO1 may have context dependent or tissue specific roles on OA susceptibility in injury vs aging.

Our genetic and functional analyses suggest that normal PIEZO1 activity is necessary to maintain joint homeostasis in both weight-bearing and non-weight-bearing joints. This is consistent with rodent studies demonstrating a role for PIEZO1 in both weight-bearing joints (knee) (86, 87, 105) and non-weight-bearing joints (temporomandibular joint) (102, 104, 106, 107). Individuals with familial hypomorphic *PIEZO1* mutations develop EHOA or IP joint OA in the absence of injury or hyperphysiological load. While these families were identified based on the EHOA or IP joint OA phenotypes, in almost all cases, affected individuals have OA in additional joints, including OA of the weight-bearing joints (Table 1). This can be the same joint or vary between individuals in a family. This variation may depend on other physiological or environmental risk factors such as patient activity level, chronic inflammation, or other factors that may alter joint loading and PIEZO1 activity (18, 108). Our data is in apparent contrast to previous *in vitro* studies, indicating that PIEZO1 has a limited role under normal loading conditions (17, 18, 19, 20, 21). Based on our genetic and *in vitro* electrophysiological analyses, we propose that PIEZO1 has important roles in the normal response to loading in addition to the well-characterised PIEZO1 response to acute injury or hyperphysiological loading. Hyperphysiological activation of PIEZO1 in cartilage induces inflammatory and catabolic gene expression, cell death, and senescence (3, 17, 18, 19, 20, 21, 24), which supports the hypothesis that activation of PIEZO1 in the joint promotes the development of OA in response to injury. Recent transcriptomic data examining mechanical activation of PIEZO1 in chondrocytes are consistent with previously published data, supporting a central role of PIEZO1 in hyperphysiological load and promoting OA-associated gene expression (23). This study also discovered unexpected pathways regulated by chemical activation of PIEZO1 in the absence of mechanical load. A two-week treatment of chondrocytes with the chemical agonist Yoda1, resulted in expression of anabolic and prochondrogenic pathways and an increase of sulfated glycosaminoglycans. Furthermore, activation of PIEZO1 in the knee joints using Yoda1 protects against injury-induced OA in the mouse (29). In sum, our human genetic data, *in vitro* electrophysiological data, and gene expression studies indicate that PIEZO1 might have context-dependent activity in homeostasis vs acute or traumatic injury. In line with our hypothesis, several mutant mouse models of OA have context-specific effects, including the *Trpv4* mutant (109, 110), altering susceptibility to post-traumatic OA and age-dependent OA in different directions (111, 112, 113).

A functional PIEZO channel assembles as a trimer. It is not yet proven (as per structural and biochemical analysis) whether PIEZO channels exist as hetero-trimers or if the PIEZO1/2 hetero-trimer can form a functional channel. It has been reported that dominant hypomorphic alleles *PIEZO1* associated with left ventricular outflow tract obstructions act as dominant negatives (68). Our results are consistent with this data. Our electrophysiology single-channel data indicated that all four OA-associated familial PIEZO1 mutations function as dominant-negative channels, with a strong possibility of forming hetero-trimeric channels with WT subunit. This dominant-negative effect may be a common mechanism shared among dominant hypomorphic alleles of *PIEZO1* (68).

There are >25 inherited mutations (both recessive and dominant and gain- and loss-of-function) in *PIEZO1* associated with various human diseases (5, 61, 114), yet our studies are the first to show a clear association with increased OA susceptibility. We do not understand why individuals with the OA-associated familial mutations specifically develop OA. There are several possibilities. Individuals with reduced PIEZO1 function may have a decrease in compensatory mechanisms that maintain joint homeostasis over time or alteration of PIEZO1 activity in the joint may disrupt the normal cellular response to everyday use or minor injury, leading to increased OA development during aging. It is plausible that chondrocytes (or a subset of chondrocytes or other cells in the joint) are exquisitely sensitive to these mutations. Three of the mutations (p.R1398W, p.K2528R and p.P2536L) cluster together on the intercellular side of the protein. These mutations may disrupt tissue-specific protein-protein interactions necessary for normal PIEZO1 activity. We know that there are many genetic, physical and environmental risk factors that contribute to OA susceptibility (36, 39, 115). There may be specific risk factors (*e*.*g*., obesity, exposure to environmental factors, or common genetic OA susceptibility variants) shared among family members that in combination with *PIEZO1* alleles contribute to increased OA susceptibility. While we cannot exclude this possibility, it is unlikely given that the *PIEZO1* alleles appear to be completely penetrant in our OA families.

We note that two of the *PIEZO1* alleles we discovered in the OA families have been reported in individuals diagnosed with dehydrated hereditary stomatocytosis (DHS) (62, 63, 64, 65, 66, 67) (p.K2502R and p.P2510L) and left ventricular outflow tract obstructions (LVOTO) (68) (p.K2502R). *PIEZO1* alleles associated with DHS are dominant gain-of-function and thus it is unlikely that affected individuals in the OA families segregating the p.K2502R and p.P2510L mutations would have DHS. The studies that reported p.K2502R and p.P2510L mutations associated with DHS did not perform functional studies (64, 65, 67). Furthermore, it was noted that one patient harboring the p.K2502R mutation also carried a mutation in SEC23B, which is associated with congenital dyserythropoietic anemia type II (67) and the two individuals carrying the p.P2510L mutation had additional mutations in *PIEZO1* (p.G2433R and p.V598L) (65). The p.K2502R mutation is one of three *PIEZO1* mutations (p.Y2022H and p.S217L) identified in a cohort of individuals diagnosed with LVOTO (68). Similar to our work, Faucherre *et*. *al*. overexpressed WT or mutant PIEZO1 in HEK cells and analyzed the electrophysiological properties of the channels. Their data demonstrates that all three *PIEZO1* mutations significantly reduced mechano-stimulated currents (68). These results are consistent with our data and indicate that the p.K2502R and p.P2510L mutations are a loss-of-function and likely do not contribute to the DHS phenotype. These data highlight the importance of single-channel functional studies on candidate disease alleles.

Given the association of the p.K2502R mutation with LVOTO, it is not surprising that the affected individuals in the OA families have phenotypes associated with this disease (Table 1). These phenotypes include atrial fibrillation, heart murmur, or sinus node dysfunction (69, 70, 71, 72). Unfortunately, it was not reported whether any of the individuals diagnosed with LVOTO also had OA phenotypes (68). Individuals segregating the p.R1404W mutation also have atrial fibrillation, heart murmur, or sinus node dysfunction (Table 1), suggesting that these dominant loss-of-function alleles can contribute to other PIEZO1-associated phenotypes, even if they are not completely penetrant.

The combined cellular, molecular, and genetic data suggest the role of PIEZO1 and PIEZO2 in joint homeostasis, and OA may be more complex than previously appreciated. *Piezo1* null mice are embryonic lethal, and the role of PIEZO1 in articular chondrocytes remains unclear (4, 116). Deletion of *Piezo1* and *Piezo2* (using *Gdf5-Cre*) in mouse cartilage has no measurable effect on injury-induced OA (105). This is in direct contrast to a recent study indicating that deletion of *Piezo1* and *Piezo2* (using *Acan-Cre*) in mouse cartilage protects against injury-induced cartilage damage and pain sensitivity, while loss of *Piezo2* protected against injury-induced bone remodeling (87). These data are consistent with a study showing deletion of *Piezo1* (using *Col2a-Cre*) protected against injury-induced OA (86). A recent study demonstrated that PIEZO1 has cell-type-specific function in tibial Procr + superficial chondrocytes. Deletion of *Piezo1* in these cells resulted in exacerbation of injury-induced OA development (29), indicating that *Piezo1* activity in these cells is needed to protect against injury-induced OA. These studies are in direct conflict with those that deleted *Piezo1* and *Piezo1 and Piezo2* more broadly in articular chondrocytes. Together, the mouse data indicate that *Piezo1* and possibly *Piezo2* have tissue and cell-type-specific roles in the joint.

It is difficult to determine the primary contribution of PIEZO1 to OA in several of these studies. The *Piezo1*^*Col2a1Cre*^ mice have defects in endochondral ossification and other bone phenotypes, all of which may contribute to OA development independent of PIEZO1 function in mature articular chondrocytes (86). Loss of *Piezo2* in articular cartilage has a protective effect on bone remodeling and indicates crosstalk between these two tissues during the onset or progression of OA (87). The use of spatially restricted null alleles of *Piezo1* (or *Piezo2*) in the context of acute injury may not reflect the contribution of the channels in the whole joint during injury or spontaneous age-associated OA. Furthermore, the deletion of *Piezo1* in a subset of articular chondrocytes has opposite effects on OA development compared with the deletion of *Piezo1* in all chondrocytes, indicating cell-type-specific roles for PIEZO1 (29, 86, 87). PIEZO1 (and PIEZO2) may have context-dependent and tissue-specific roles in other tissues of the joint, such as synovium, immune cells, or the infrapatellar fat pad, which would not be uncovered by used cartilage specific deletion of *Piezo1* (117, 118). For example, nociceptor-specific deletion of *Piezo2* (using *NaV1*.*8-Cre*) protects against mechanical sensitization while having no effect on cartilage degeneration and osteophyte formation in response to injury-induced OA (119). While our data helps refine the role of PIEZO1 activity in OA, we clearly need better *in vivo* models to address the potential context-dependent roles of PIEZO1 in different cell types of the synovial joint. Generation of mice with human-specific *PIEZO1* OA alleles will be important for determining how alteration of PIEZO1 channel activity contributes to disease onset and progression in the context of injury vs aging (37).

In sum, we have discovered rare *PIEZO1* coding mutations associated with familial age-associated erosive hand OA (EHOA) or interphalangeal (IP) joint OA. Our functional analysis indicates that these mutations act as dominant-negative alleles to reduce the open probability of the channel. Furthermore, we demonstrate that the *PIEZO1* GWAS allele associated with reduced OA progression has increased channel activity. The familial and GWAS alleles have differential effects on gene expression in two independent cell types *in vitro*. Given the genetic and functional data, we propose that PIEZO1 likely has context and tissue-dependent effects in injury-induced vs. age-associated OA.

## Experimental procedures

### Study approval

The Institutional Review Board of the University of Utah and the Resource for Genetic and Epidemiologic Research approved this study. Written informed consent was obtained under the guidance of the Institutional Review Board of the University of Utah (IRB # 00079442). Animal studies were approved by the University of Utah Institutional Animal Care and Use Committee (IACUC # 00001786).

### Sex as a biological variable

Sex was not considered as a biological variable. Our study exclusively utilized human cells from a female donor and mouse cells isolated from female mice.

### Diagnostic and procedure codes used to identify individuals with osteoarthritis

Our coding strategy used to identify individuals with EHOA and distal and proximal interphalangeal joint OA has been previously described (37, 39). Briefly, the following diagnostic codes (ICD-9 and ICD-10) and procedure codes (CPT - Current Procedural Terminology) were used to identify affected individuals. EHOA: ICD-10 M15.4 (Erosive (osteo)arthritis). Distal and proximal interphalangeal joint OA: CPT: 26862, 26863, 26860, 26861 (arthrodesis, interphalangeal joint, with or without internal fixation) and 26535, 26536 (arthroplasty, interphalangeal joint). ICD-9: 715.14 (osteoarthritis, localized, primary, hand). ICD-10: M19.04x (primary osteoarthritis, hand). Individuals diagnosed with any of the following codes were excluded: ICD-9: 714.0 (rheumatoid arthritis), 714.2 and 714.3 (rheumatoid arthritis and other inflammatory polyarthropathies). ICD-10: M05.xxx (rheumatoid polyneuropathy with rheumatoid arthritis), M06.xx (other rheumatoid arthritis), or M08.xxx (juvenile arthritis). Individuals were asked if they were diagnosed with psoriatic arthritis, gout, or had a traumatic or acute injury to the affected joint. If they answered yes, they were excluded from the study.

### Whole exome sequencing and analysis

Families with a statistically significant increase in incidence of OA that appeared to segregate as a simple dominant Mendelian trait were identified and selected for whole exome sequencing (WES) (36, 37, 38). WES and analysis was performed using genomic DNA isolated from whole blood or saliva as previously described (38). Briefly, libraries were prepared using the Agilent SureSelect XT Human All Exon + UTR (v8) kit followed by Illumina NovaSeq 6000 150 cycle paired end sequencing. We followed best practices established by the Broad Institute GATK for variant discovery (https://gatk.broadinstitute.org/hc/en-us). Analysis of variants was performed with ANNOVAR (http://annovar.openbioinformatics.org/en/latest/) (120) and pVAAST (http://www.hufflab.org/software/pvaast/) (121) in concert with PHEVOR2 (http://weatherby.genetics.utah.edu/phevor2/index.html) (122). pVAAST is a probabilistic search tool that classifies variants with respect to the likely effect on gene function. It incorporates information, including the position of a variant, cross-species phylogenetic conservation, biological function, and pedigree structure. PHEVOR2 works in concert with the output of pVAAST to integrate phenotype, gene function, and disease information for improved power to identify disease-causing alleles.

### PIEZO1 protein alignment

We utilized the UCSC Genome Browser (https://genome.ucsc.edu/) centered on Human Assembly GRCh38/hg38 to examine amino acid conservation across multiple vertebrates. Conservation was visualized using the Vertebrate Multiz Alignment & Conservation using the default species reading frames for translation.

### Electrophysiology

Transfected HEK293T^ΔP1^ cells (ATCC CRL-3519) were visualized using Nikon eclipse Ti2 microscope and C11440 Orca-Flash 4.0 LT digital camera (Hamamatsu) to identify PIEZO1 or mutant’s expressing green cells (GFP tagged) for single construct transfection, or PIEZO1 expressing red cells (TdTomato tagged) and mutants expressing green cells (GFP tagged) for co-expression studies. Cell-attached recordings of pressure-activated currents in HEK293T^ΔP1^ cells expressing the WT PIEZO1, OA-associated familial and GWAS mutations were performed using Axopatch 200B amplifier and Digidata 1550B digitizer (Molecular Devices). Currents were acquired with pClamp 10.7 software at a sampling frequency of 10 kHz and filtered at 2 kHz. Recording patch pipettes of borosilicate glass (Sutter Instruments BF150–86–10) were pulled and fire-polished to a tip resistance of 4 to 6 MΩ. The bath solution contained (in mM): 140 KCl (Fisher Bioreagents, BP366–500), 10 HEPES (Gibco, 15630080), 1 MgCl_2_ (Millipore Sigma, 63069), 10 glucose (Sigma, G8270-1 KG), pH 7.3 (with KOH (LabChem LC193702), Osmolarity = 280 ± 1 mOsm (n = 3). The pipette solution contained (in mM): 130 NaCl (Fisher Brand, S271–3), 5 KCl, 10 HEPES, 10 TEA-Cl (Acros Organics, 150901000), 1 CaCl_2_ (Millipore Sigma, 21115), 1 MgCl_2_ pH 7.3 (with NaOH), Osmolarity = 287 ± 1.5 mOsm (n = 3). When mentioned, 20 μM Yoda1 (Tocris, 5586/10) was added in the pipette solution. Mechanical stimulation was applied by using a high-speed pressure clamp system (ALA Scientific Instruments). Single-channel events are shown as downward deflection (inward currents) acquired at −60 mV (to match conventional direction of ion flow). The data was analyzed using Clampfit 10.7. The Open Probability was calculated by: Po = *A*o/(*A*c + *A*o). A_O_ and Ac are the areas under the curve of open and closed components of all-point histograms respectively. The areas under the curve of histograms were calculated using Clampfit program and *via* Gaussian fits the data.

### PIEZO1 clone generation

Much of the functional data on PIEZO1 has been generated using the mouse protein, including Cryo EM structures and electrophysiological data. Therefore, we utilized the mouse cDNA sequences in our functional studies. The mouse PIEZO1-GFP fused gene was synthesized using GenScript’s Clone EZ service. OA point mutations were generated from this parent clone, also using GenScript’s Clone EZ. For calcium flux assay, a Myc tag was introduced following amino acid 2422 and GFP was deleted to avoid fluorescence signal from fused fluorophore. All clones were sequence verified after every maxi prep DNA isolation. Primers for sequencing were as follows:

R537C (forward): 5′- CTGGTGGTCCTGTCACTTTC -3′

R1398W (forward): 5′- GGGACTGCCTCATCCTCTATAA -3′

K2528R and P2536L and 2422-Myc tag for all mutants, used the following primers:

(forward): 5′- TTCCCCATCTCTTCCCCAAG -3′

(reverse): 5′- GGAAGATGAGCTTGGCGTATAG -3′

### Cell culture and transient transfection

Method for calcium flux experiments is as previously published (46). Briefly, adherent *PIEZO1* knock-out cells (HEK293T^ΔP1^) were harvested with 0.05% trypsin/1xEDTA and plated onto poly-D-lysine (Gibco)-coated 96-well black-sided/clear-bottom plates (Costar 3603) at sufficient density to achieve near-confluency after 24 h. Cells were plated in 1xDulbecco’s modified Eagle’s medium (DMEM) supplemented with 10% fetal bovine serum (FBS; Sigma) and 1x GlutaMAX (Gibco), Osmolarity = 332 ± 4 mOsm (n = 3), and modified manufacturer’s protocols used to transiently transfect cells using Lipofectamine 3000 (ThermoFisher). Cells were incubated 24 h at +37 °C and 8% CO2 to allow for adherence and expression prior to calcium flux assays. For single-channel recordings, cells were cultured as described above, plated onto poly-D-lysine (Gibco)-coated glass coverslips at least 2 h before transfection. HEK293T^ΔP1^ cells were transfected with 1 μg of PIEZO1-GFP, p.R537C-GFP, p.R1398W-GFP, p.K2528R-GFP, p.P2536L-GFP and p.F2484L-GFP and PIEZO1-TdTomato constructs, using Lipofectamine 3000 (ThermoFisher) according to the manufacturer’s instructions. For each construct, 6 to 9 individual transfections were performed on separate days, and cells from 4 to eight coverslips from each transfection were used to collect single-channel records. All the recordings were performed between 18 and 26 h after transfection to obtain single-channel data. Cell lines were tested and were negative for *mycoplasma*.

### Subconductance state, occupancy, and mean lifetime analysis

The occupancies and lifetimes of various conducting states are calculated as previously described (73). Briefly, to process the 20 kHz unfiltered data, a Butterworth low-pass filter was applied with a cut-off frequency of 20 Hz to each trace. QuB was used to apply a baseline at the shut state without compromising open states. The data is idealized under the Viterbi algorithm with shut, open and sub-conductance states in QuB. The idealized data is used to find occupancy and mean lifetime, and to generate dwell time histograms. The current amplitude histograms were plotted on a log scale and fitted with a four exponentials-function *via* the least square method.

### Calcium flux assay

Fluo-4 AM NW dye and probenecid (Molecular Probes) were prepared according to manufacturer’s instructions with the reaction buffer containing 1xHBSS (Gibco 14175079) + 4 mM CaCl_2_ +1 mM MgCl_2_, Osmolarity = 296 ± 0.5 mOsm (n = 3). Yoda-1 (Tocris) was prepared as 10 mM stock in 100% DMSO (Molecular Probes). HEK293T (ATCC CRL-3216), HEK293T^ΔP1^ (ATCC CRL-3519) and TC28a2 chondrocytes (Millipore Sigma SCC042) were seeded in a 96-well plate and reverse-transfected. After 24 h incubation at +37 °C and 8% CO_2,_ cells were loaded with dye/probenecid for 40 min at +37 °C and 8% CO_2_. After loading cells with dye, baseline fluorescence was measured for 5 min followed by an 18-min measurement of activation with 20 μM Yoda1 (Tocris)/0.2% DMSO (Molecular Probes) or 0.2% DMSO alone. A23187 calcium ionophore was added, and cells assayed for 25 min to measure the maximum cellular calcium signal. Calcium flux was assayed on a BioTek Synergy H1 at +37 °C (E x 488/Em518) with 70 s time points.

### Primary human chondrocyte cell culture

Primary human chondrocytes (CELLvo HC-a (passage 1)) were obtained from StemBioSys, Inc. CELLvo Chondrocytes were isolated from cadaveric knee articular cartilage of a healthy 28-year-old female African American donor. CELLvo HC-a chondrocytes were cultured on CELLvo ChondroMatrix-coated plasticware (StemBioSys, Inc.) in low-glucose Dulbecco's Modification of Eagle's Medium (DMEM) (Corning) supplemented with 1 g/L glucose, 1 mM sodium pyruvate, 4 mM L-glutamine, 15% fetal bovine serum and 100 U/ml penicillin/streptomycin in a 5% CO_2_ humidified atmosphere at 37 °C as previously described (45).

### Electroporation of primary human chondrocytes

Electroporation of CELLvo HC-a chondrocytes was carried out with the Neon Transfection System using the 10 μl Kit (ThermoFisher Scientific) following the manufacturer’s protocol. Briefly, 200 × 10^3^ CELLvo HC-a chondrocytes and 2ug of mouse *Piezo1*^*WT*^ or *PIEZO1*^*R1398W*^ plasmids suspended in buffer R were electroporated using parameters the following parameters: 1600 V, 10 msec, 5 pulses. These conditions resulted in >95% electroporation efficiency as assayed by the total number of GFP^+^ cells. After 24 h, electroporation media was replaced with fresh low-glucose DMEM media supplemented with 1 g/L glucose, 1 mM sodium pyruvate, 4 mM L-glutamine, 15% fetal bovine serum and 100 U/ml penicillin/streptomycin containing 10 ng/ml IL1β protein (ThermoFisher Scientific) for an additional 24 h. After incubation cells were collected by trypsinization, RNA was isolated and analyzed by RT-qPCR as described below. Utilization of the mouse cDNAs allowed us to quantify *Piezo1* expression levels without interference from the endogenous human locus (see Fig. S7).

### Primary mouse synovial fibroblast isolation and culture

Synovial fibroblasts were isolated from 16-week-old C57BL/6J mice based on the protocol described in (11, 90). Briefly, mice were euthanized, and the hind paws were cleaned with proviodine rinsed with 70% EtOH. The claws and skin were removed, and a scalpel was used to generate longitudinal cuts between the toes. Dissected paws were digested in high glucose DMEM (Gibco) containing 400 μg/ml Collagenase IV (Sigma), 400 μg/ml Lberase (Sigma), and 400 μg/ml DNaseI (Sigma) and incubated at 37 ^o^C for 45 to 50 min. Samples were vortexed at high speed for 10 s every 5 min. The digested sample was filtered using a 40 μm cell strainer, centrifuged at 500*g* for 5 min and resuspended in DMEM, 10% v/v FBS and 1X antibiotic/antimycotic solution (Penicillin G, Streptomycin, Amphotericin B (HyClone). Cells were transferred to a T-25 tissue culture flask. All experiments were conducted using passage three synovial fibroblasts.

### Transfection of primary mouse synovial fibroblasts

Synovial fibroblasts were transfected using Lipofectamine 3000 (ThermoFisher Scientific). Briefly, synovial fibroblasts were transfected with 0.5 μg of mouse *Piezo1*^*WT*^, *Piezo1*^*R1398W*^, or *Piezo1*^*F2484L*^ plasmid per well. Cells were cultured for 24 h, the media was replaced with control media or media containing 10 ng/ml IL1β protein (ThermoFisher Scientific) and cultured for an additional 24 h. After IL1β treatment total RNA was isolated and analyzed by RT-qPCR as described below.

### Quantitative reverse transcription PCR (RT-qPCR)

Electroporated primary CELLvo HC-a chondrocytes or transfected primary mouse synovial fibroblasts were lysed using Trizol (ThermoFisher Scientific), and total RNA was collected using the Direct-zol RNA Miniprep Kit (Zymo Research). RNA quantity and purity was assessed by Nanodrop 2000 (ThermoFisher). Total of 50 ng RNA was combined with PrimeTime One-Step RT-qPCR Master Mix (IDT) and predesigned gene-specific assays (IDT, PrimeTime qPCR Probe Assays) and RT-qPCR was performed according to the manufacture’s protocol. Gene expression was normalized to *ACTB*, and relative expression was calculated using the ΔΔCq method.

### Statistics

All-point histograms of the single-channel data (from 15 to 30 s stretch) were constructed in Clampfit. The single-channel current values were extracted after fitting the Gaussian function to the data. Each mean value is an average of at least nine or at most 27 individual patch recordings. 8 to 12 individual coverslips with transfected cells, either WT PIEZO1 or mutants, were used to acquire the data. The open probability of the channel was calculated exclusively from the records, where at least 10 s of data were recorded both before and after pressure application. The number of channels in the patch is determined by fitting a Gaussian curve to all-point current histograms for individual open peak. In cases where more than one channel was observed (2 or three channel record detectable by simultaneous openings) the open probability was adjusted for a single channel. Group data (bar graphs) are presented as Mean ± Standard Deviation, where ∗ *p* < 0.05; ∗∗*p* < 0.01, ∗∗∗*p* < 0.001, ∗∗∗∗*p* < 0.0001, not significant (ns) *p* > 0.05. Unpaired two-tailed t-tests were used for comparison between the two groups (WT and mutant channels). One-way ANOVA Post-Hoc test (Bonferroni correction) was performed for multiple comparisons. For RT-qPCR experiments, mRNA levels are reported as fold-change (mean ± SEM) relative to *Piezo1*^*WT*^ electroporation. Statistically significant differences as described above were determined by a two-tailed unpaired *t* test or a two-way ANOVA with Tukey’s multiple comparisons test, n = 3 or 4 biological replicates as indicated in the scatter plots.

## Data availability

Data is available upon request. Restrictions apply to the availability of the genomic and medical data analyzed in this study to preserve patient confidentiality. Access to UPDB data is controlled through the Resource for Genetic and Epidemiologic Research.

## Supporting information

This article contains supporting information.

## Conflict of interest

The authors declare that they do not have any conflicts of interest with the content of this article.
