# Supplementary material for: PIEZO1 variants that reduce open channel probability are associated with familial osteoarthritis
Source: J Biol Chem. 2026 Apr 2;302(5):111426. doi: 10.1016/j.jbc.2026.111426 (PMC13157073; doi:10.1016/j.jbc.2026.111426)
Supplement: Supplementary Material — 1 [file mmc1.pdf]

Supporting Information

PIEZO1 variants that reduce open channel probability are associated with familial osteoarthritis

Michael J. Jurynec[1,2,3]*, Elena Nosyreva[4]*, David Thompson[4], Kendra A. Novak[1], Derek J. Matheson[1], Shivakumar R. Veerabhadraiah[1], Ying Ma[1], Matthew C Smith[1], Nikolas H. Kazmers[1], Stefan Feier[5], Ghanim Ullah[5] and Ruhma Syeda[4]

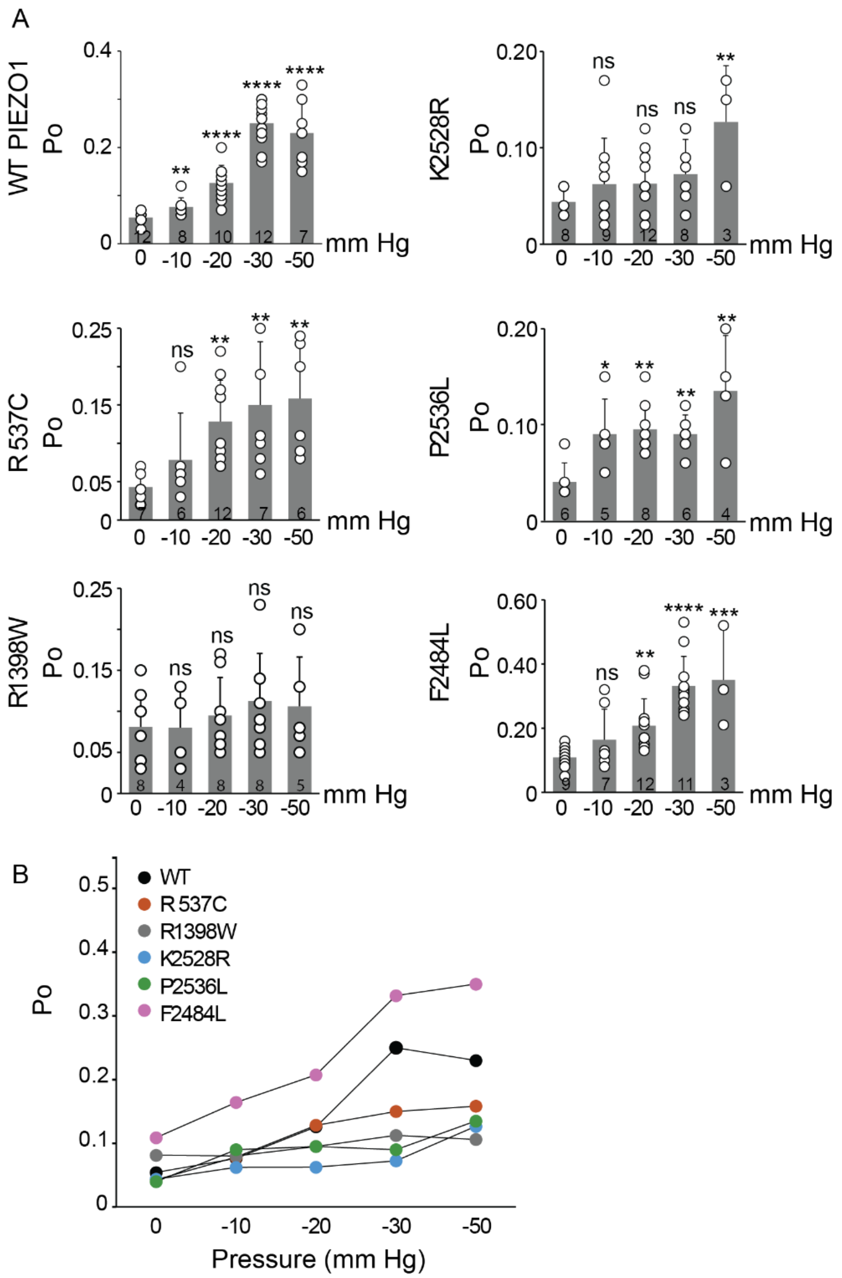

Supplemental Figure 1. Single Channel Open probability (Po) of WT PIEZO1 and Indicated OA-Associated Mutants at Various Pressures. (A) Comparison of open probability before (zero pressure) and after indicated pressures (-10 to -50 mm Hg). **(B)** Mean Po acquired from panel A, presented on the same plot, to indicate loss-of-function for familial mutants (p.R537C, p.R1398W, p.K2528R, p.P2536L) and gain-of-function for GWAS mutant p.F2484L when compared to WT at various pressures. Unpaired two tail t test, ∗p < 0.05, ∗∗p < 0.01, ∗∗∗p < 0.001, ****p<0.0001 and ns = not significant. Experimental replica numbers are shown in the bar graphs.

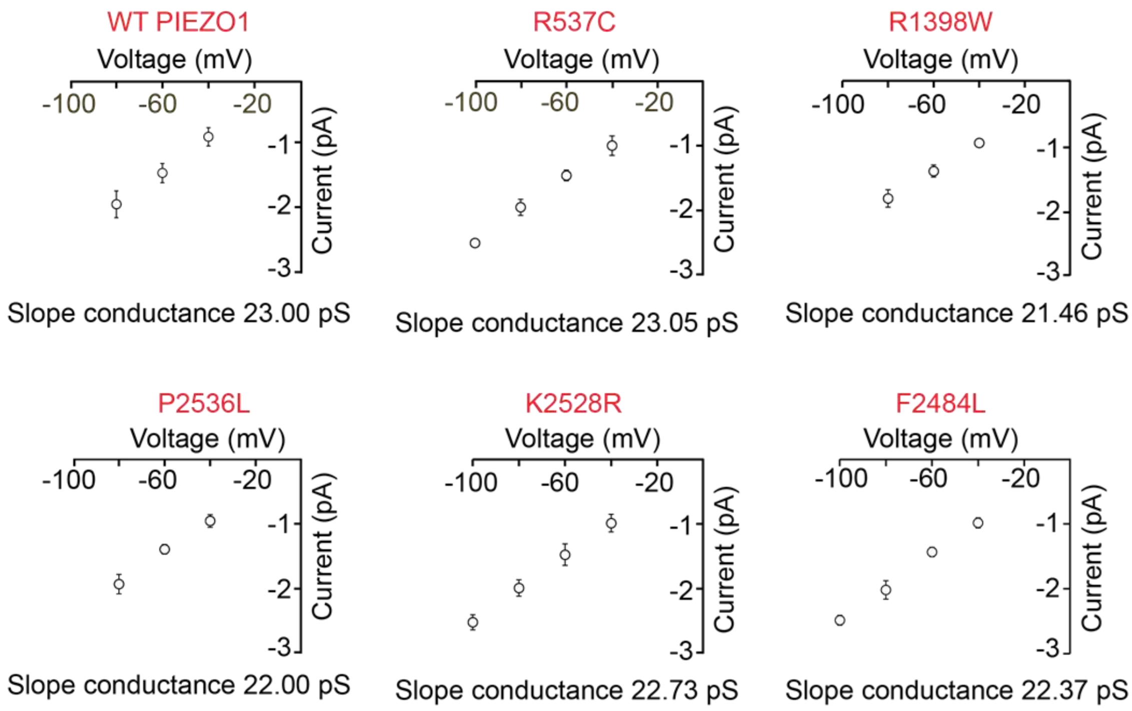

Supplemental Figure 2. Current Voltage relationship of WT PIEZO1, OA-associated familial mutations (p.R537C, p.R1398W, p.K2528R, and p.P2536L) and the GWAS mutation p.F2484L, with indicated slope conductance. No significant difference was observed between WT and mutant's slope conductance ($p>0.05$). Experimental replica (n) > 7.

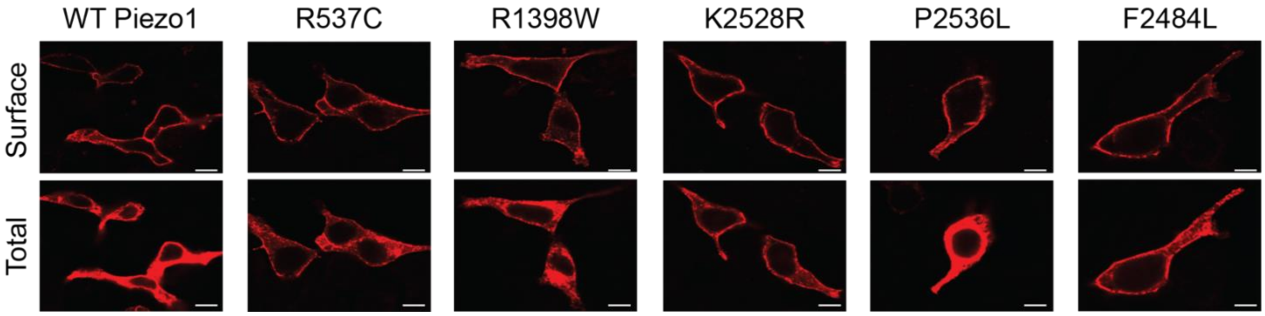

Supplemental Figure 3. Surface Expression of WT and OA-associated Mutant Channels. Representative confocal images of Myc labeling (n=5) in HEK293T$^{\Delta P1}$ cells expressing WT PIEZO1 and OA-associated mutants. Myc tag was introduced in the cap domain of PIEZO1 at 2422 amino acid position. Immunostaining was done on fixed cells, with no permeabilization (Surface, upper row), staining only the surface proteins. Afterwards, the cells were permeabilized and stained, staining all of the myc-tagged PIEZO1 within the cell (Total, bottom row). The positive staining of all the construct and qualitative analysis suggests that dysfunctional PIEZO1 is trafficked to the membrane. Scale bar, 10 µm.

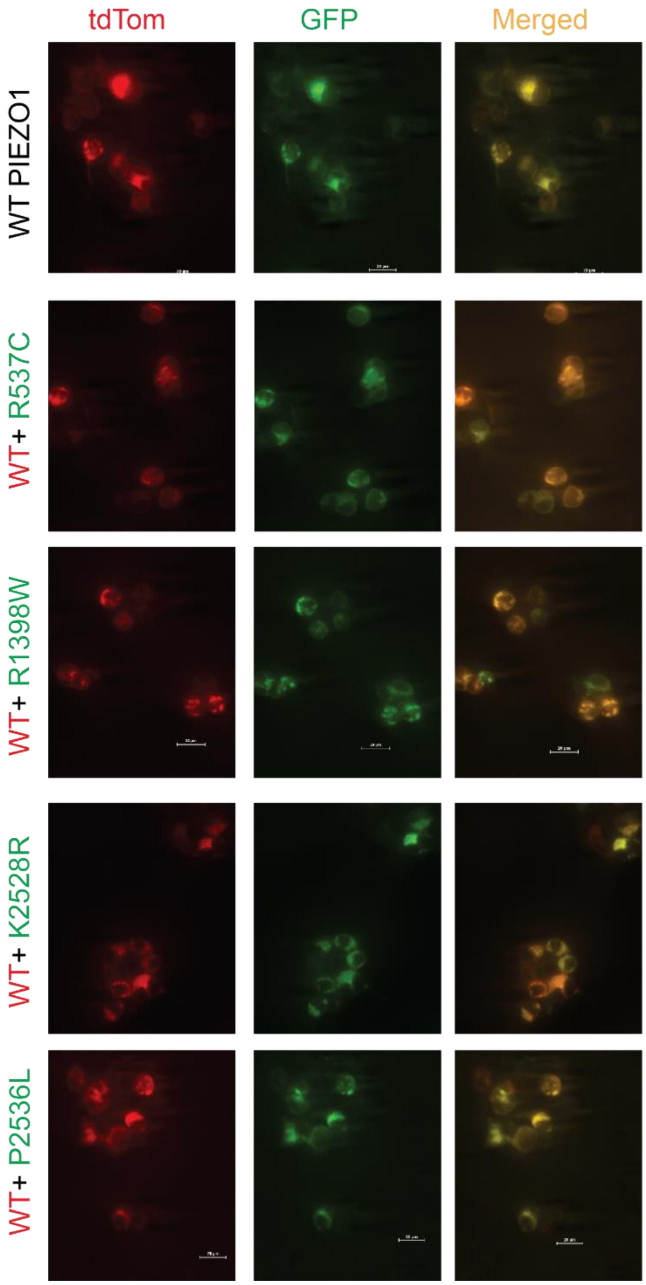

Supplemental Figure 4. Co-expression of WT PIEZO1 fused with TdTomato and indicated mutants fused with GFP. Cells exhibiting uptake of both WT and mutant construct were used for patching and obtaining data for Figure 4. Scale bar is 20 µm. Images were captured using Nikon eclipse Ti2 microscope and C11440 Orca-Flash 4.0 LT digital camera (Hamamatsu).

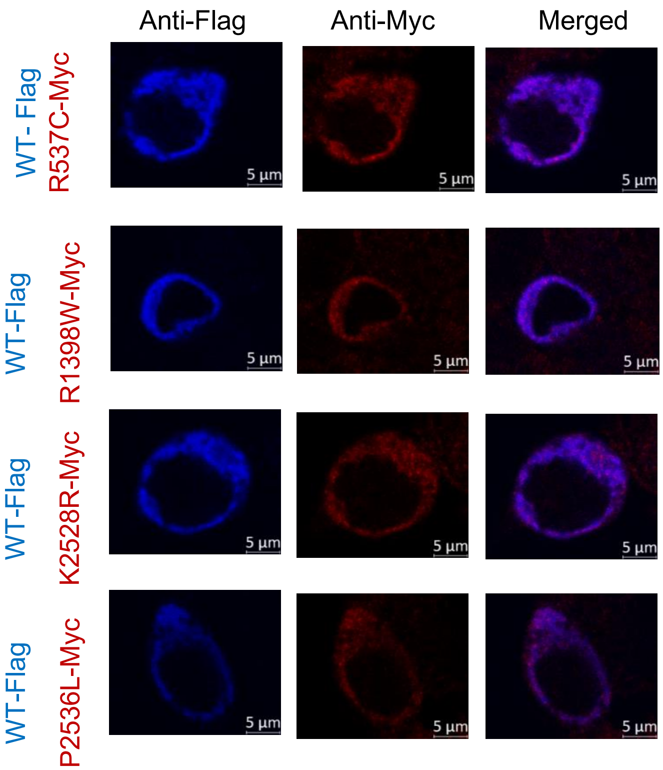

Supplemental Figure 5. Confocal Images of Co-expressed WT and OA-associated Mutant Channels. Coexpression and colocalization of WT PIEZO1 with Flag tag at extracellular amino acid 2422, and indicated mutants with Myc tag at amino acid 2422. Representative confocal images of Flag and Myc labeling in HEK293T$^{\Delta P1}$ cells expressing WT PIEZO1 and OA-associated mutants. Immunostaining was done on fixed cells, without permeabilization, staining only the surface proteins. The positive staining of all the construct and qualitative analysis suggests that both the WT and mutant PIEZO1 are trafficked to the membrane. Images were captured with Zeiss LSM880 at 63x, oil immersion. Scale bar, 5 µm.

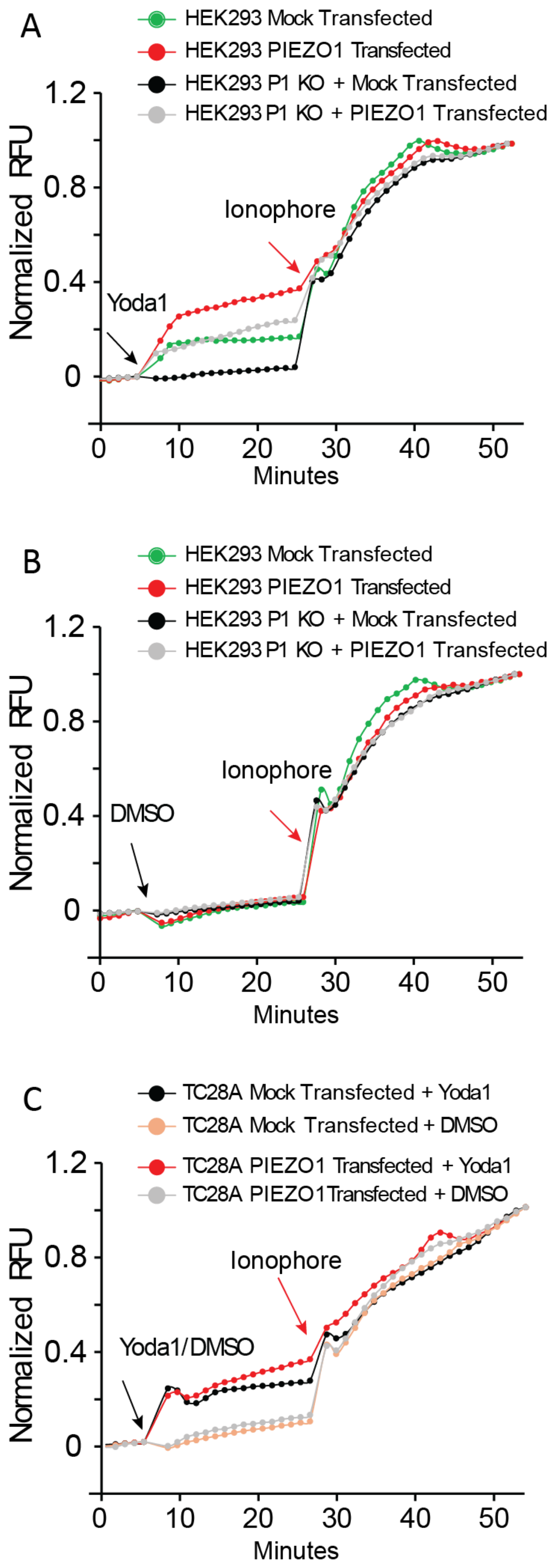

Supplemental Figure 6. Yoda1 Stimulation of Calcium Influx in Cultured Cells. PIEZO1 activity of endogenous or heterologous expression in HEK293, HEK293$^{\Delta P1}$, and TC28a2 chondrocyte cells, as measured by intracellular calcium concentration and fluorescent detection. Relative fluorescence of each individual well is normalized by setting baseline background fluorescence (before addition of Yoda1 or DMSO control) as 0, and maximum fluorescence (after addition of calcium ionophore A23187) as 1. Data points/curves represent normalized fold-stimulation of the maximum activated signal prior to the addition of A23187 ionophore relative to DMSO control. SD error bars for panels (A - C) not shown for clarity. Additions of Yoda1 or DMSO are indicated by black arrows, Ionophore A23187 by red arrows. Cells were pre-loaded with Fluo-4 dye and probenecid before treatment with 20µM Yoda1 or 0.2% DMSO control. **(A)** Yoda1 induces calcium influx in Mock-transfected endogenously-expressing HEK cells but not in Mock-transfected PIEZO1 null cells. Transient transfection of endogenously-expressing HEK293 cells increases normalized RFU 2.2-fold over mock-transfection, while transfection of PIEZO1 null cells increases signal 6-fold over mock. (**B)** DMSO alone does not stimulate calcium influx in any of the cell lines, regardless of PIEZO1 endogenous or heterologous expression. (**C**) Yoda1 induces calcium influx in both Mock- and PIEZO1-transfected TC28a2 cells. Transfection of TC28a2 with PIEZO1 minimally increases calcium-flux signal over mock-transfection. DMSO alone does not stimulate calcium influx in both transfections of TC28a2 cells. Calcium flux of TC28a2 fails to achieve steady-state plateau under the conditions tested. Experimental replica (n) > 10.

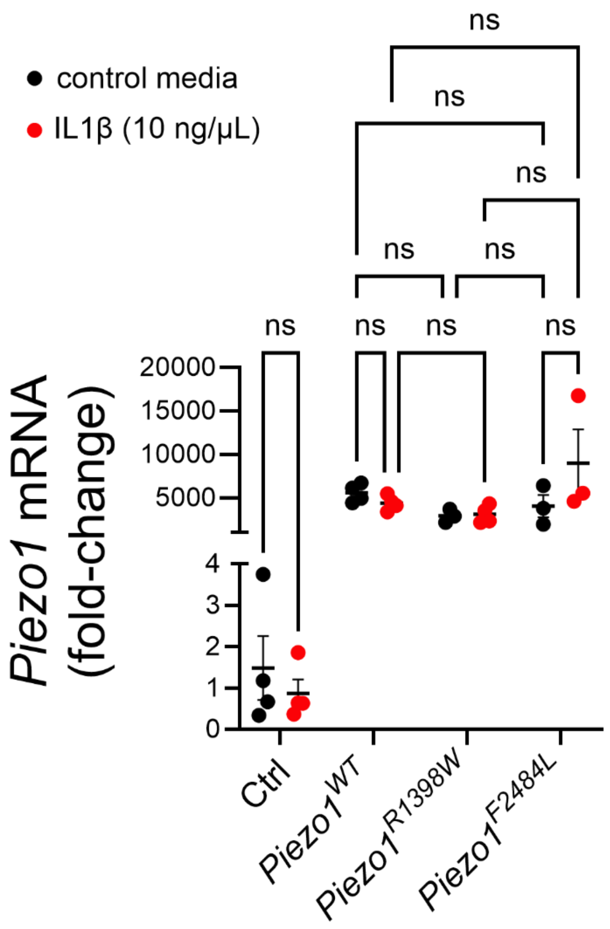

Supplemental Figure 7. RT-qPCR analysis on primary human chondrocytes expressing *Piezo1*^{WT}, *Piezo1*^{R1398W}, or *Piezo1*^{F2484L} under control conditions or IL1β treatment. RT-qPCR analysis of mouse *Piezo1* gene expression in control electroporated primary human chondrocytes (Ctrl) or primary human chondrocytes electroporated with mouse plasmids encoding *Piezo1*^{WT}, *Piezo1*^{R1398W} , or *Piezo1*^{F2484L}. Gene expression in chondrocytes treated with control media (black circles) or chondrocytes treated with IL1β (10 ng/ml) for 24 hours (red circles). Gene expression was normalized to *ACTB* and relative expression was calculated using the ΔΔCq method. mRNA levels are reported as fold-change (mean ± SEM) relative to control electroporated primary human chondrocytes. Statistically significant differences were tested by a two-way ANOVA with Tukey's multiple comparisons test, n=3 or 4 biological replicates. ns = not significant.

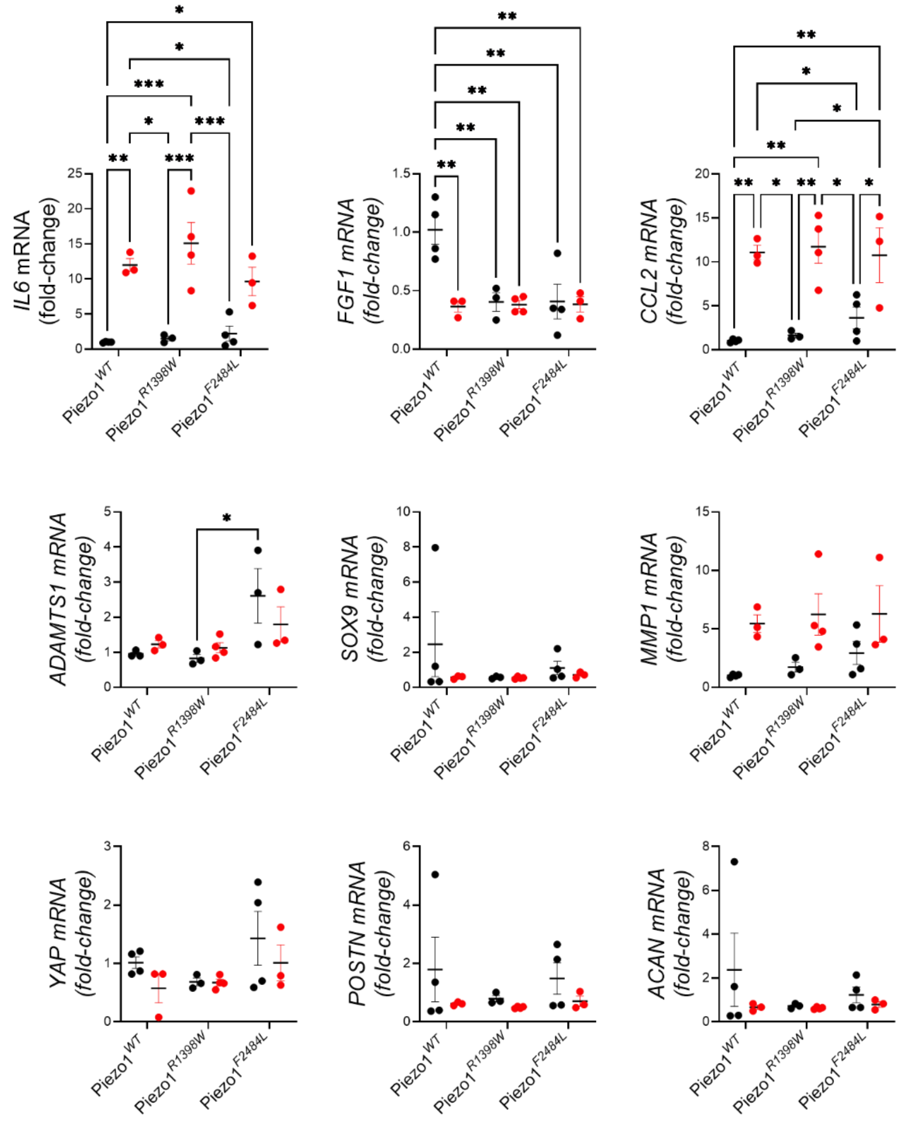

Supplemental Figure 8. Primary human chondrocytes respond to IL1β treatment by upregulation of OA-associated genes. RT-qPCR analysis of gene expression in primary human chondrocytes electroporated with *Piezo1*^{WT}, *Piezo1*^{R1398W}, or *Piezo1*^{F2484L}. Gene expression in untreated chondrocytes or (black circles) or chondrocytes treated with IL1β (10ng/ml) for 24 hours (red circles). Gene expression was normalized to *ACTB* and relative expression was calculated using the ΔΔCq method. mRNA levels are reported as fold-change (mean ± SEM) relative to *Piezo1*^{WT} electroporation. Statistically significant differences of $p \leq 0.05$ (*), $p \leq 0.01$ (**), and $p \leq 0.001$ (***) were determined by a two-way ANOVA with Tukey's multiple comparisons test, n=3 or 4 biological replicates.

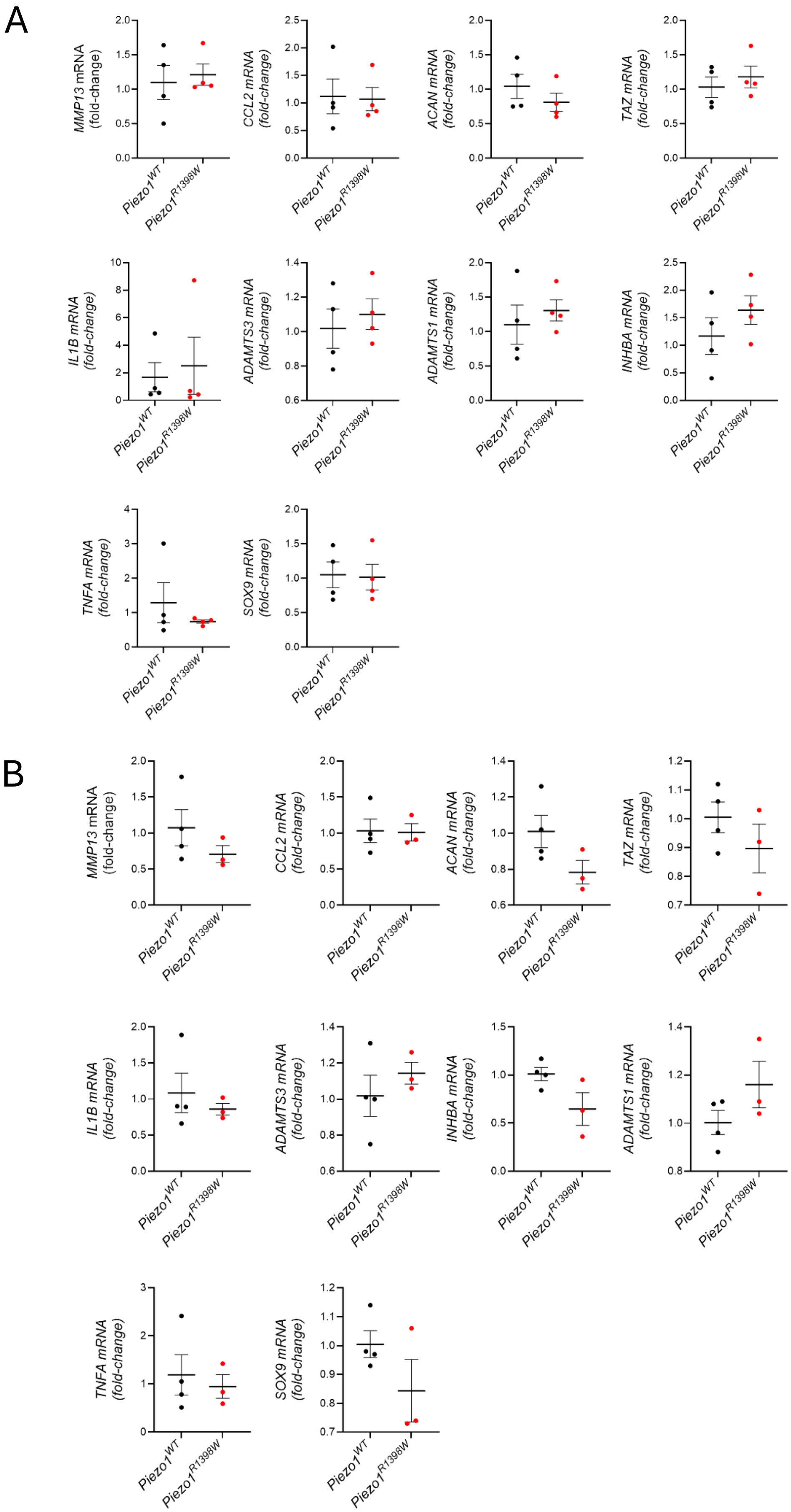
A
MMP13 mRNA (fold-change)
CCL2 mRNA (fold-change)
ACAN mRNA (fold-change)
TAZ mRNA (fold-change)
IL1B mRNA (fold-change)
ADAMTS3 mRNA (fold-change)
ADAMTS1 mRNA (fold-change)
INHBA mRNA (fold-change)
TNFA mRNA (fold-change)
SOX9 mRNA (fold-change)
Piezo1^WT
Piezo1^R1398W
B
MMP13 mRNA (fold-change)
CCL2 mRNA (fold-change)
ACAN mRNA (fold-change)
TAZ mRNA (fold-change)
IL1B mRNA (fold-change)
ADAMTS3 mRNA (fold-change)
INHBA mRNA (fold-change)
ADAMTS1 mRNA (fold-change)
TNFA mRNA (fold-change)
SOX9 mRNA (fold-change)
Piezo1^WT
Piezo1^R1398W

C

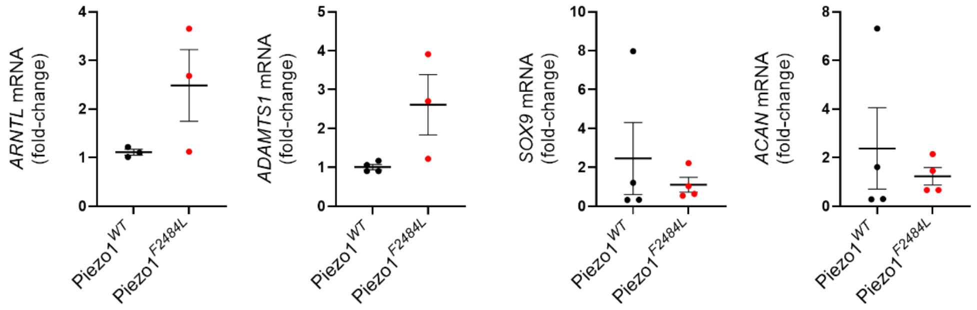

D

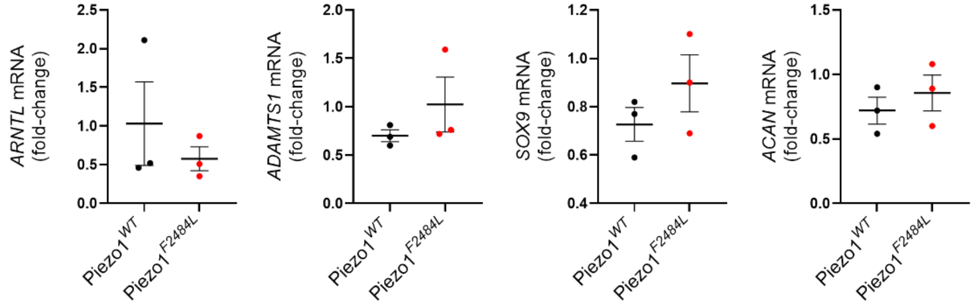

Supplemental Figure 9. RT-qPCR analysis on primary human chondrocytes expressing *Piezo1*^{WT}, *Piezo1*^{R1398W}, or *Piezo1*^{F2484L} under control conditions or IL1β treatment. RT-qPCR analysis of gene expression in primary human chondrocytes electroporated with *Piezo1*^{WT}, *Piezo1*^{R1398W}, or *Piezo1*^{F2484L}. Gene expression in untreated chondrocytes or (**A and C**) or chondrocytes treated with IL1β (10ng/ml) for 24 hours (**B and D**). **A** and **B** indicate chondrocytes expressing *Piezo1*^{WT} or *Piezo1*^{R1398W} and **C** and **D** indicate chondrocytes expressing *Piezo1*^{WT} or *Piezo1*^{F2484L}. Gene expression was normalized to *ACTB* and relative expression was calculated using the ΔΔCq method. mRNA levels are reported as fold-change (mean ± SEM) relative to *Piezo1*^{WT} electroporation. No statistically significant differences were observed using a two-tailed unpaired t-test, n=3 or 4 biological replicates as indicated in the scatter plots.

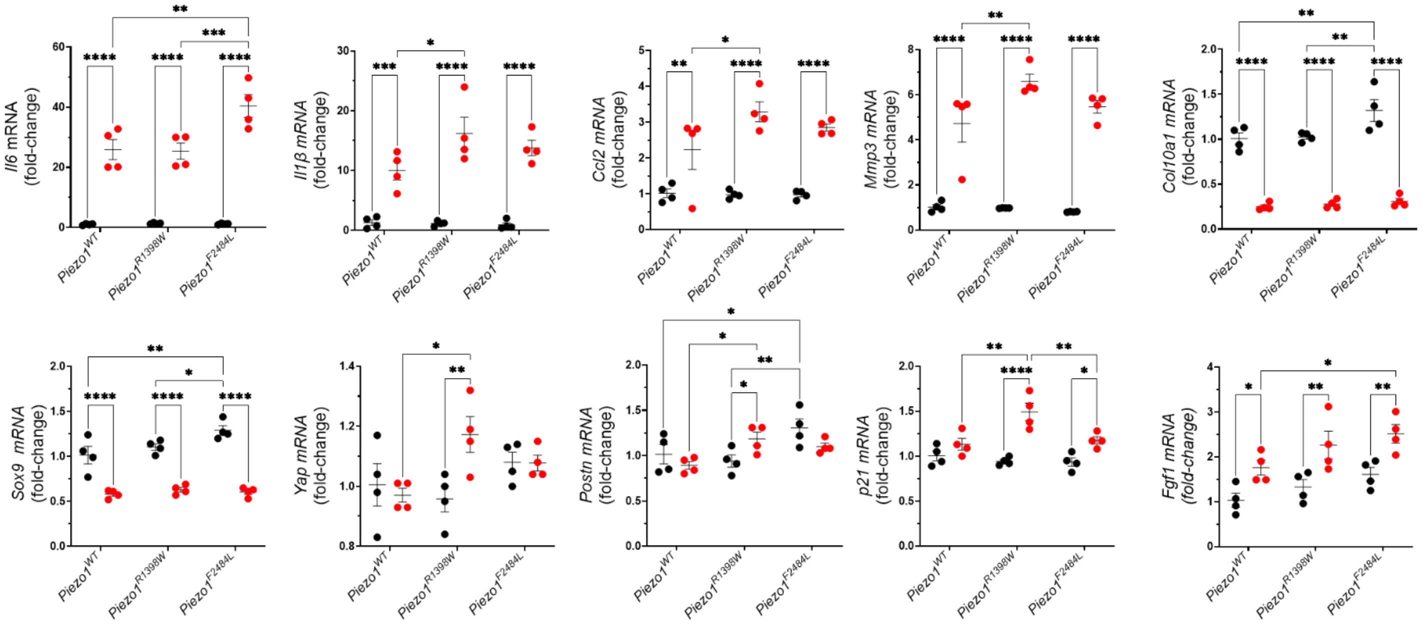

Supplemental Figure 10. Overexpression of *Piezo1*^{R1398W} or *Piezo1*^{F2484L} differentially effects the transcriptional response of primary mouse synovial fibroblasts under control conditions or IL1β treatment. RT-qPCR analysis of *Il6*, *Il1β*, *Ccl2*, *Mmp3*, *Col10a1, Sox9*, *Yap*, *Postn*, *p21*, and *Fgf1* gene expression in primary mouse paw synovial fibroblasts transfected with *Piezo1*^{WT}, *Piezo1*^{R1398W}, or *Piezo1*^{F2484L}. Gene expression in control synovial fibroblasts (black circles) or synovial fibroblasts treated with IL1β (10ng/ml) for 24 hours (red circles). Gene expression was normalized to *Actb* and relative expression was calculated using the ΔΔCq method. mRNA levels are reported as fold-change (mean ± SEM) relative to *Piezo1*^{WT} transfection. Statistically significant differences of $p \leq 0.05$ (*), $p \leq 0.01$ (**), $p \leq 0.001$ (***) and $p \leq 0.0001$ (****) were determined by a two-way ANOVA with Tukey's multiple comparisons test, n=3 or 4 biological replicates.

References

1. Jurynec, M. J., Gavile, C. M., Honeggar, M., Ma, Y., Veerabhadraiah, S. R., Novak, K. A. *et al.* (2022) NOD/RIPK2 signalling pathway contributes to osteoarthritis susceptibility Ann Rheum Dis 10.1136/annrheumdis-2022-222497
2. Kazmers, N. H., Meeks, H. D., Novak, K. A., Yu, Z., Fulde, G. L., Thomas, J. L. *et al.* (2020) Familial clustering of erosive hand osteoarthritis in a large statewide cohort Arthritis Rheumatol 10.1002/art.41520
3. Song, S., Zhang, H., Wang, X., Chen, W., Cao, W., Zhang, Z. *et al.* (2022) The role of mechanosensitive Piezo1 channel in diseases Prog Biophys Mol Biol **172**, 39-49 10.1016/j.pbiomolbio.2022.04.006
4. Gavile, C. M., Kazmers, N. H., Novak, K. A., Meeks, H. D., Yu, Z., Thomas, J. L. *et al.* (2022) Familial Clustering and Genetic Analysis of Severe Thumb Carpometacarpal Joint Osteoarthritis in a Large Statewide Cohort J Hand Surg Am 10.1101/2022.03.03.22271851
5. Jurynec, M. J., Sawitzke, A. D., Beals, T. C., Redd, M. J., Stevens, J., Otterud, B. *et al.* (2018) A hyperactivating proinflammatory RIPK2 allele associated with early-onset osteoarthritis Hum Mol Genet **27**, 2406 10.1093/hmg/ddy196
6. Wang, K., Li, M., andHakonarson, H. (2010) ANNOVAR: functional annotation of genetic variants from high-throughput sequencing data Nucleic Acids Res **38**, e164 10.1093/nar/gkq603
7. Hu, H., Roach, J. C., Coon, H., Guthery, S. L., Voelkerding, K. V., Margraf, R. L. *et al.* (2014) A unified test of linkage analysis and rare-variant association for analysis of pedigree sequence data Nat Biotechnol **32**, 663-669 10.1038/nbt.2895
8. Singleton, M. V., Guthery, S. L., Voelkerding, K. V., Chen, K., Kennedy, B., Margraf, R. L. *et al.* (2014) Phevor combines multiple biomedical ontologies for accurate identification of disease-causing alleles in single individuals and small nuclear families Am J Hum Genet **94**, 599-610 10.1016/j.ajhg.2014.03.010
9. Amado, N. G., Nosyreva, E. D., Thompson, D., Egeland, T. J., Ogujiofor, O. W., Yang, M. *et al.* (2024) PIEZO1 loss-of-function compound heterozygous mutations in the rare congenital human disorder Prune Belly Syndrome Nat Commun **15**, 339 10.1038/s41467-023-44594-0
10. Ullah, G., Nosyreva, E. D., Thompson, D., Cuello, V. A., Cuello, L. G., andSyeda, R. (2024) Analysis of pressure-activated Piezo1 open and subconductance states at a single channel level J Biol Chem **300**, 107156 10.1016/j.jbc.2024.107156
11. Knights, A. J., Farrell, E. C., Ellis, O. M., Lammlin, L., Junginger, L. M., Rzeczycki, P. M. *et al.* (2023) Synovial fibroblasts assume distinct functional identities and secrete R-spondin 2 in osteoarthritis Ann Rheum Dis **82**, 272-282 10.1136/ard-2022-222773
