# Supplementary material for: PIEZO1 variants that reduce open channel probability are associated with familial osteoarthritis
Source: J Biol Chem. 2026 Apr 2;302(5):111426. doi: 10.1016/j.jbc.2026.111426 (PMC13157073; doi:10.1016/j.jbc.2026.111426)
Supplement: Supplementary Material — 2 [file mmc2.pptx]

## Slide 1
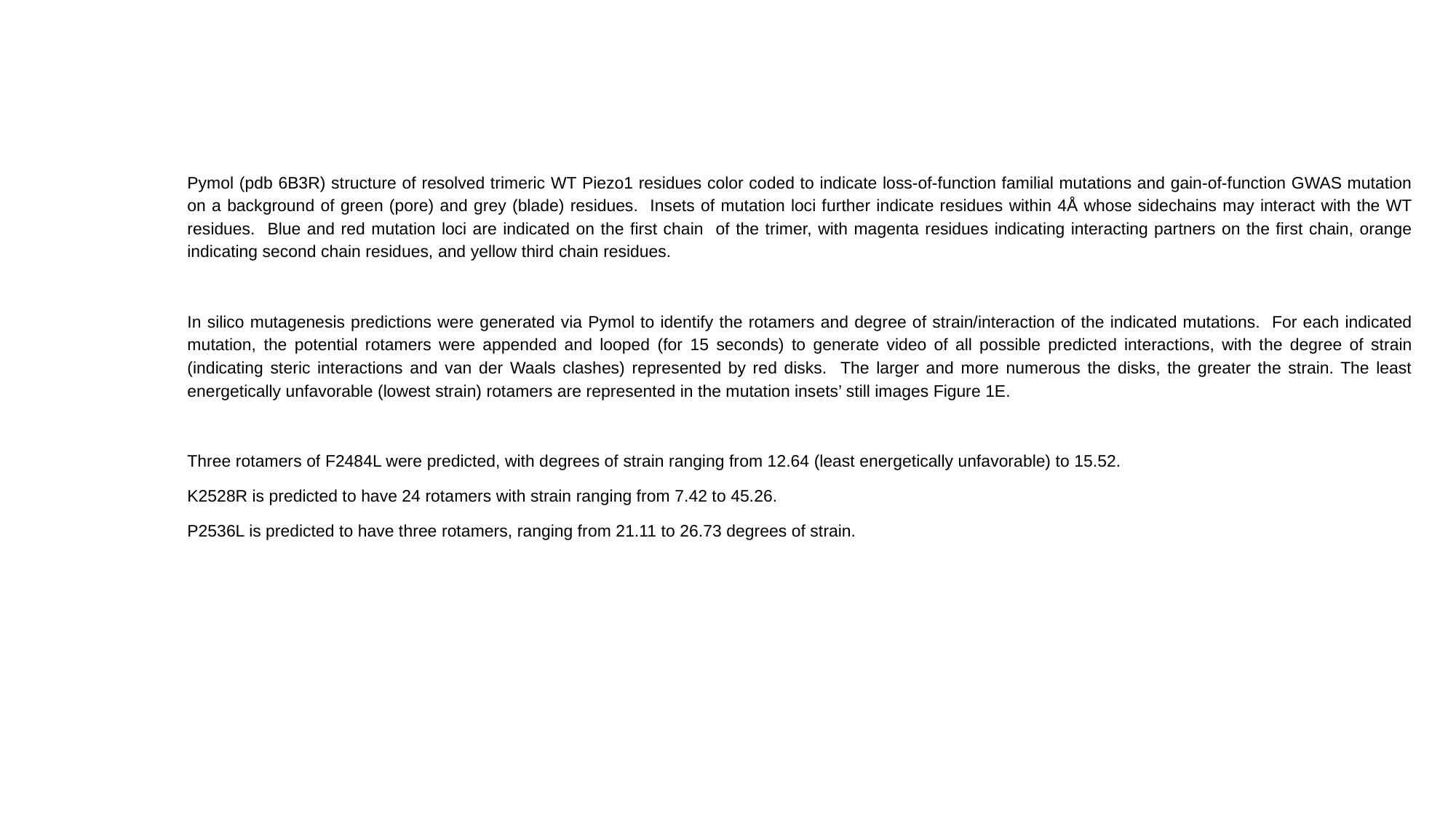

Pymol (pdb 6B3R) structure of resolved trimeric WT Piezo1 residues color coded to indicate loss-of-function familial mutations and gain-of-function GWAS mutation on a background of green (pore) and grey (blade) residues. Insets of mutation loci further indicate residues within 4Å whose sidechains may interact with the WT residues. Blue and red mutation loci are indicated on the first chain of the trimer, with magenta residues indicating interacting partners on the first chain, orange indicating second chain residues, and yellow third chain residues.
In silico mutagenesis predictions were generated via Pymol to identify the rotamers and degree of strain/interaction of the indicated mutations. For each indicated mutation, the potential rotamers were appended and looped (for 15 seconds) to generate video of all possible predicted interactions, with the degree of strain (indicating steric interactions and van der Waals clashes) represented by red disks. The larger and more numerous the disks, the greater the strain. The least energetically unfavorable (lowest strain) rotamers are represented in the mutation insets’ still images Figure 1E.
Three rotamers of F2484L were predicted, with degrees of strain ranging from 12.64 (least energetically unfavorable) to 15.52.
K2528R is predicted to have 24 rotamers with strain ranging from 7.42 to 45.26.
P2536L is predicted to have three rotamers, ranging from 21.11 to 26.73 degrees of strain.

## Slide 2
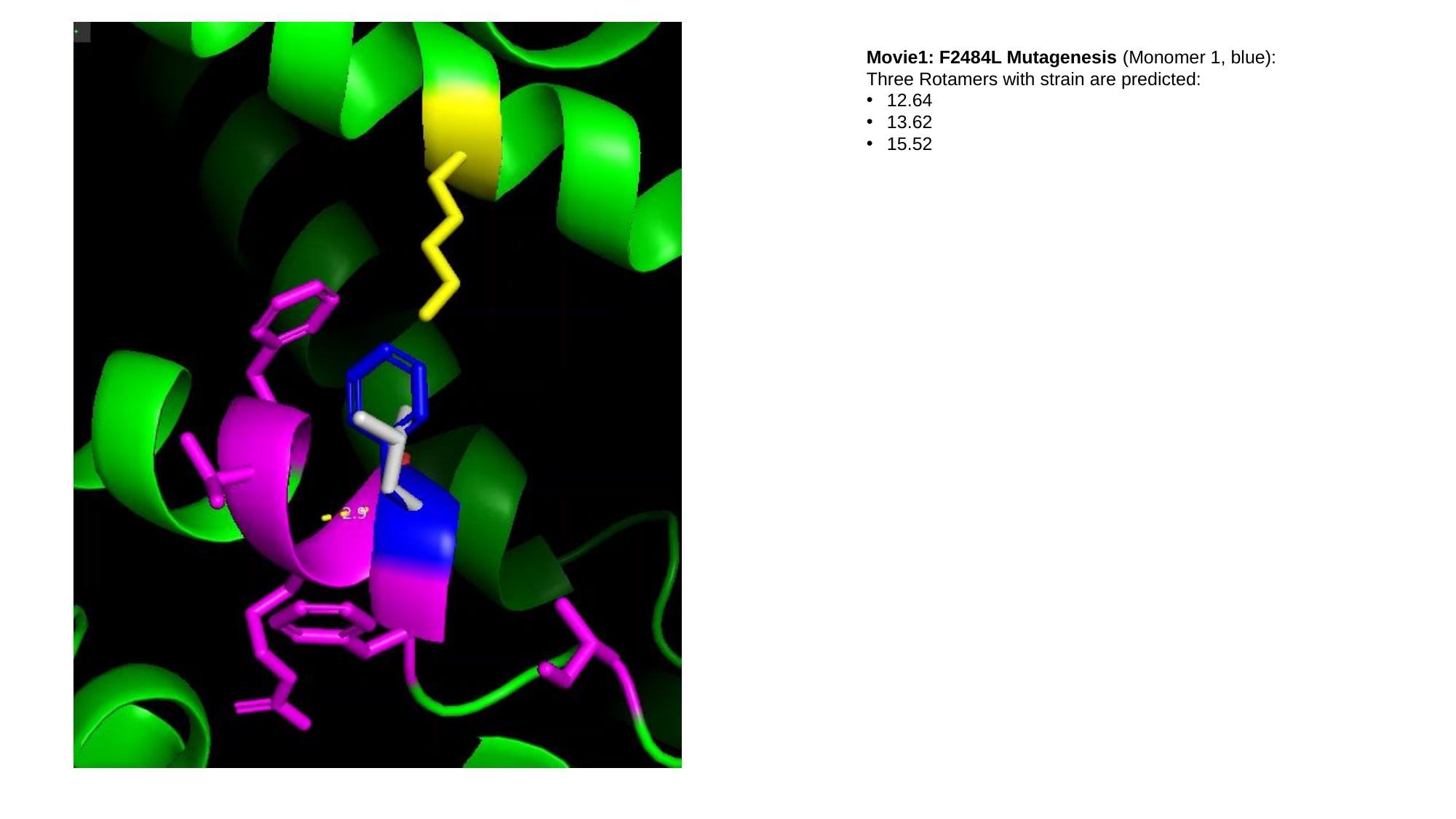

Movie1: F2484L Mutagenesis (Monomer 1, blue):
Three Rotamers with strain are predicted:
12.64
13.62
15.52

## Slide 3
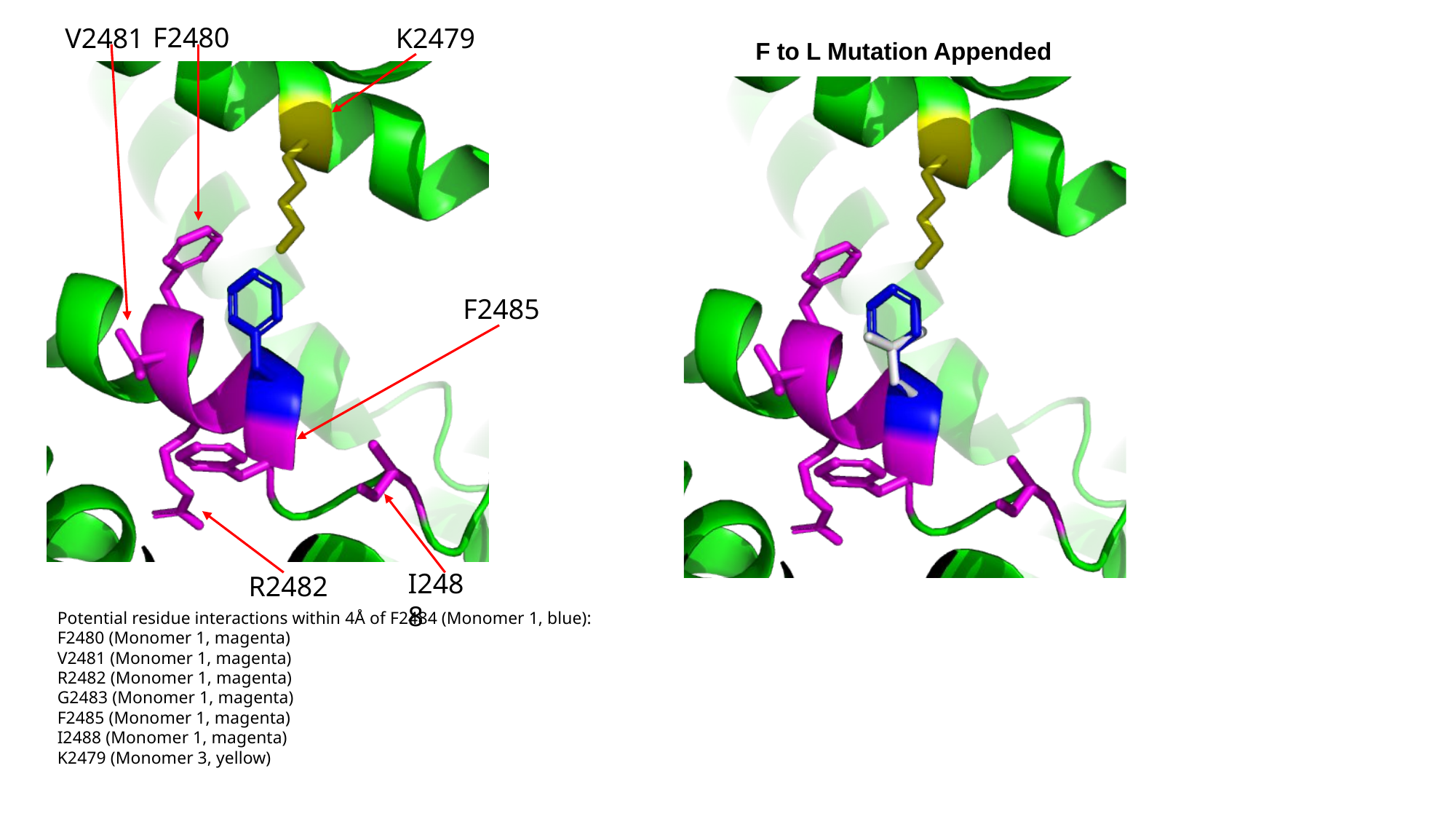

F2480
V2481
K2479
F to L Mutation Appended
F2485
I2488
R2482
Potential residue interactions within 4Å of F2484 (Monomer 1, blue):
F2480 (Monomer 1, magenta)
V2481 (Monomer 1, magenta)
R2482 (Monomer 1, magenta)
G2483 (Monomer 1, magenta)
F2485 (Monomer 1, magenta)
I2488 (Monomer 1, magenta)
K2479 (Monomer 3, yellow)

## Slide 4
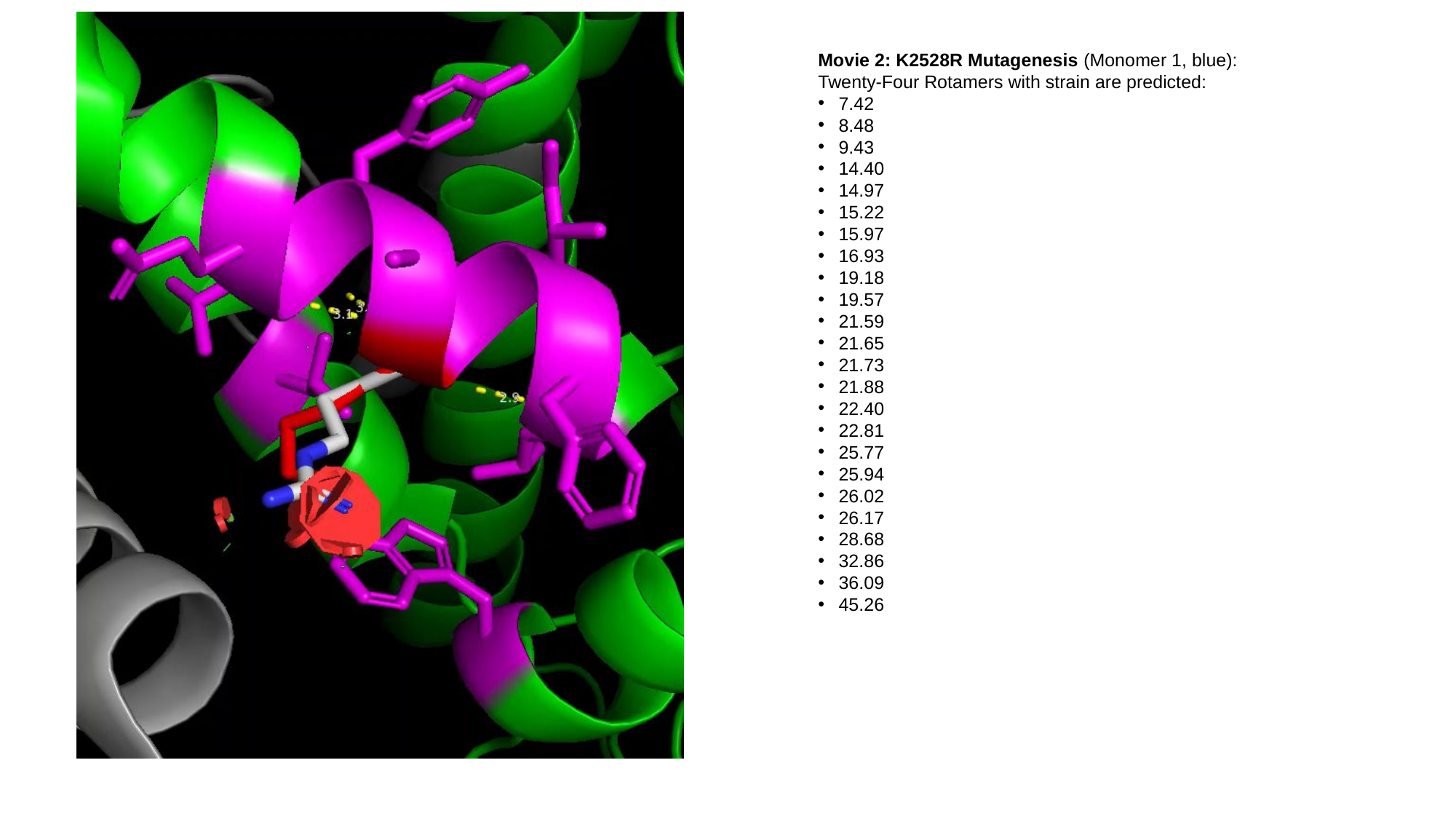

Movie 2: K2528R Mutagenesis (Monomer 1, blue):
Twenty-Four Rotamers with strain are predicted:
7.42
8.48
9.43
14.40
14.97
15.22
15.97
16.93
19.18
19.57
21.59
21.65
21.73
21.88
22.40
22.81
25.77
25.94
26.02
26.17
28.68
32.86
36.09
45.26

## Slide 5
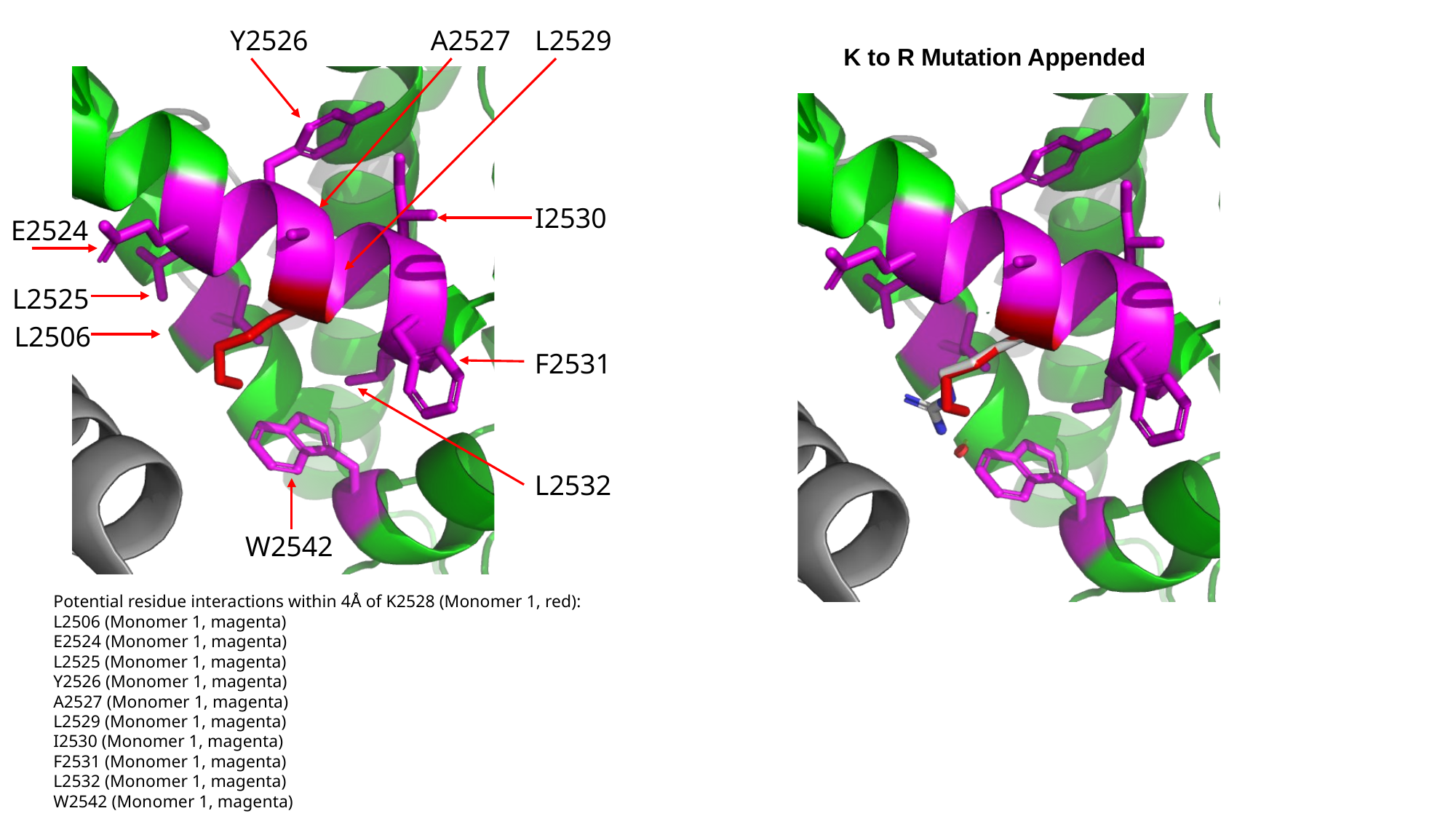

A2527
L2529
Y2526
K to R Mutation Appended
I2530
E2524
L2525
L2506
F2531
L2532
W2542
Potential residue interactions within 4Å of K2528 (Monomer 1, red):
L2506 (Monomer 1, magenta)
E2524 (Monomer 1, magenta)
L2525 (Monomer 1, magenta)
Y2526 (Monomer 1, magenta)
A2527 (Monomer 1, magenta)
L2529 (Monomer 1, magenta)
I2530 (Monomer 1, magenta)
F2531 (Monomer 1, magenta)
L2532 (Monomer 1, magenta)
W2542 (Monomer 1, magenta)

## Slide 6
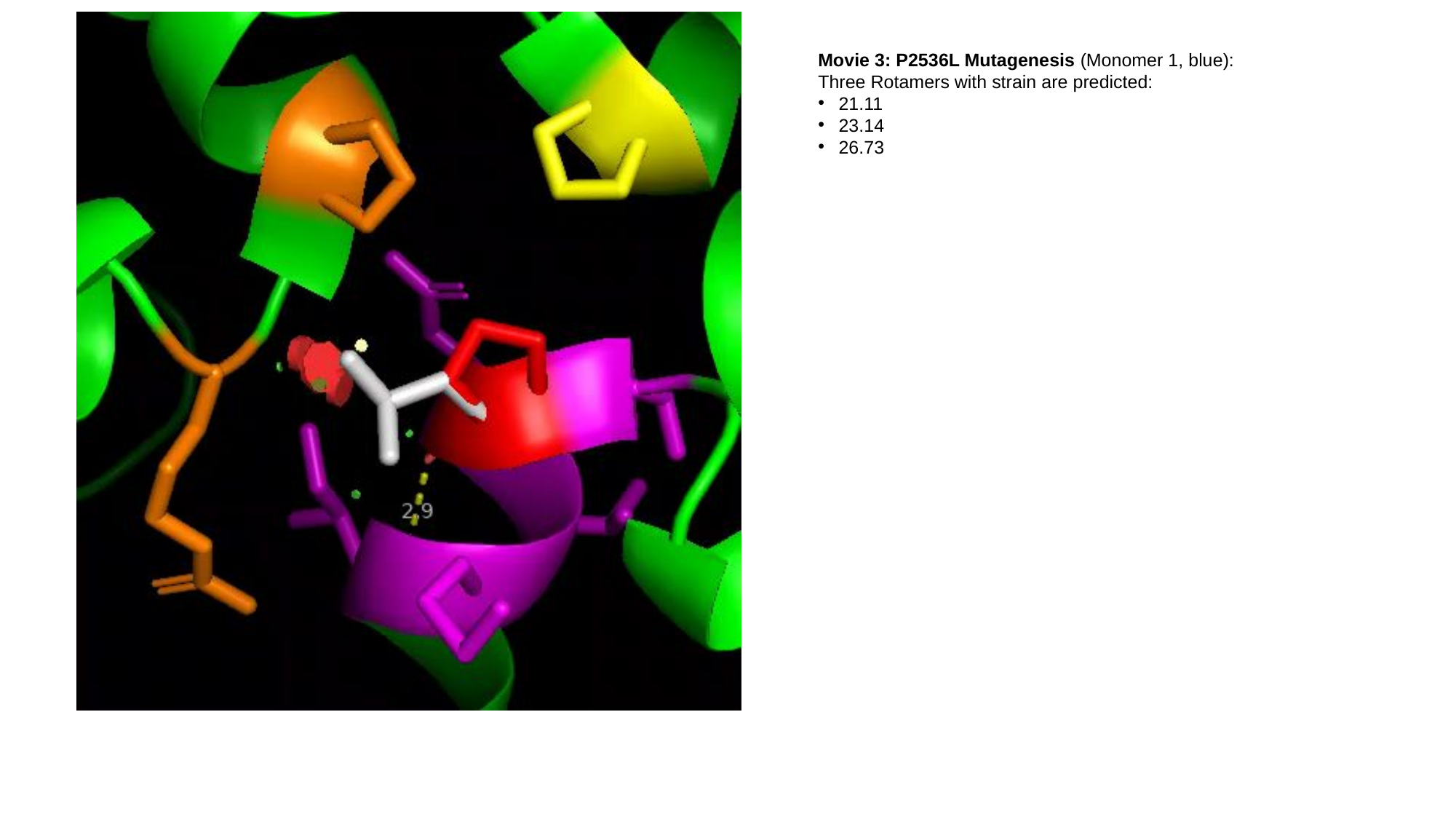

Movie 3: P2536L Mutagenesis (Monomer 1, blue):
Three Rotamers with strain are predicted:
21.11
23.14
26.73

## Slide 7
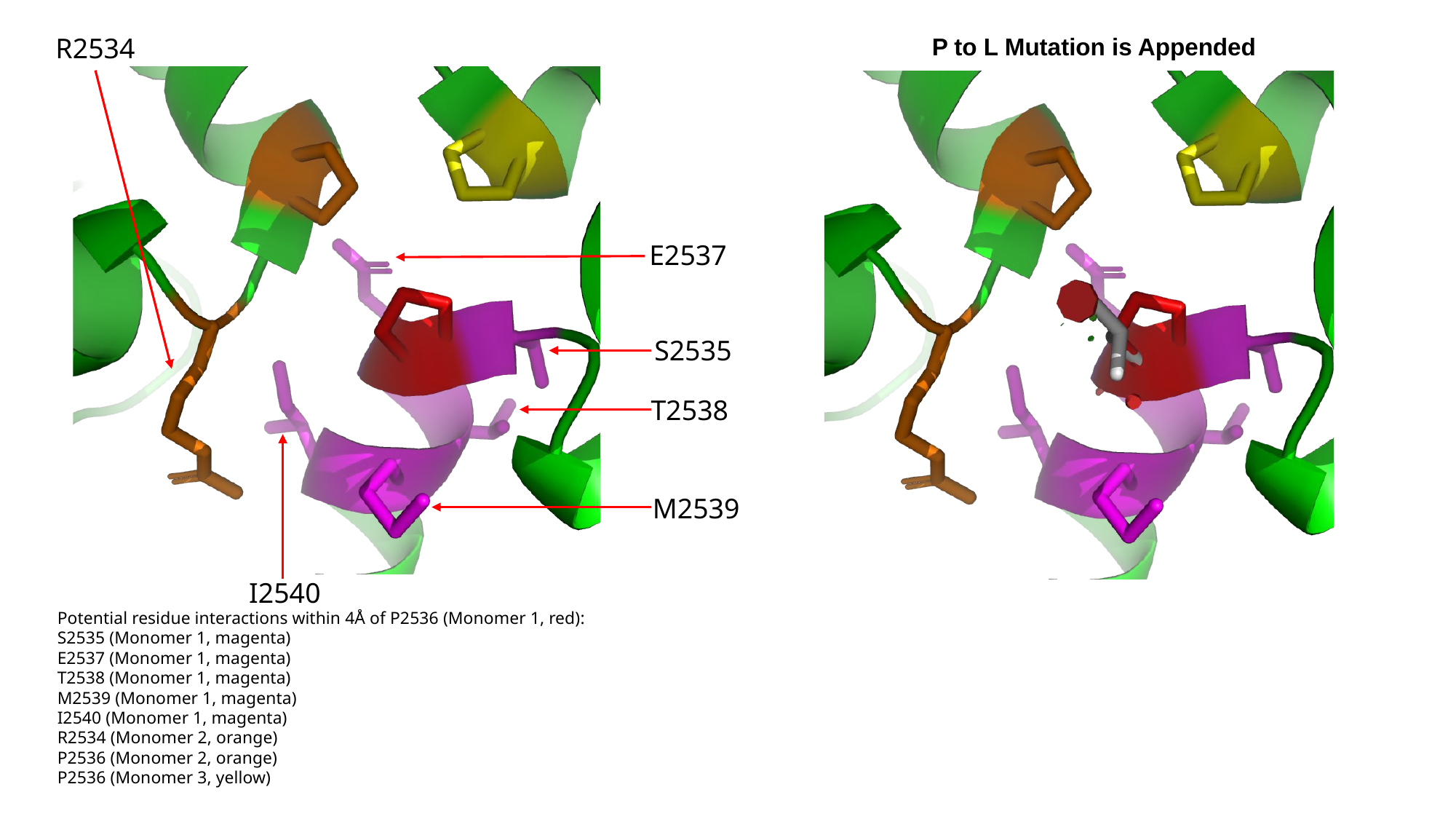

R2534
P to L Mutation is Appended
E2537
S2535
T2538
I2540
M2539
Potential residue interactions within 4Å of P2536 (Monomer 1, red):
S2535 (Monomer 1, magenta)
E2537 (Monomer 1, magenta)
T2538 (Monomer 1, magenta)
M2539 (Monomer 1, magenta)
I2540 (Monomer 1, magenta)
R2534 (Monomer 2, orange)
P2536 (Monomer 2, orange)
P2536 (Monomer 3, yellow)
